# Design, Synthesis, and Bioassay of 2′-Modified Kanamycin A

**DOI:** 10.3390/molecules27217482

**Published:** 2022-11-02

**Authors:** Ribai Yan, Xiaonan Li, Yuheng Liu, Xinshan Ye

**Affiliations:** 1 National Demonstration Center for Experimental Pharmacy Education, School of Pharmaceutical Sciences, Peking University, Beijing 100191, China; 2 State Key Laboratory of Natural and Biomimetic Drugs, School of Pharmaceutical Sciences, Peking University, Beijing 100191, China; 3 Department of Medical Chemistry, College of Pharmaceutical Science, Hebei Medical University, Shijiazhuang 050017, China

**Keywords:** antibiotic, aminoglycoside, structural modification, structure–activity relationship

## Abstract

Chemical modification of old drugs is an important way to obtain new ones, and it has been widely used in developing new aminoglycoside antibiotics. However, many of the previous modifying strategies seem arbitrary for their lack of support from structural biological detail. In this paper, based on the structural information of aminoglycoside and its drug target, we firstly analyzed the reason that some 2′-*N*-acetylated products of aminoglycosides caused by aminoglycoside-modifying enzyme AAC(2′) can partially retain activity, and then we designed, synthesized, and evaluated a series of 2′-modified kanamycin A derivatives. Bioassay results showed our modifying strategy was feasible. Our study provided valuable structure–activity relationship information, which would help researchers to develop new aminoglycoside antibiotics more effectively.

## 1. Introduction

In order to fight against drug-resistant bacteria, researchers need to constantly develop new antibiotics. A feasible way to acquire new antibiotics is to modify the old ones. Structural modification on old drugs not only offers new chemical entities, but also affords valuable information about the relationship between molecular structure and its activity. As a kind of clinically important antibiotic, aminoglycosides are also facing the problem of resistance. The most common mechanism for their resistance is that the bacteria acquire the capability to produce aminoglycoside-modifying enzymes [1,2,3]. After enzymic modification, the resulting products decrease or lose their affinity to the drug target and then decrease or lose their antibacterial activity. To tackle this problem, proactive structural modification on these drugs have been extensively investigated [4,5,6,7]. Through chemical modification, some sensitive groups on the drug molecules are eliminated or masked. Thus, the resulting products no longer are the proper substrates for the modifying enzymes, and then the activity remains.

To modify aminoglycosides, various chemical strategies have been developed, such as modifying the aminoglycoside core structure, developing aminoglycoside-heteroconjugates, and introducing various alkyl/aryl substituents and acyl substituents at different positions on aminoglycoside scaffolds [8]. However, in the view of the mutual interaction between drug and drug target, some of them seem arbitrary. Ribosomal 16S RNA A-site of bacteria is the pivot for codon recognition during protein synthesis. It is established that most aminoglycosides bind to this domain in a specific mode and then exhibit antibacterial activity [9,10,11,12]. Obviously, this specific drug–target interaction mode should be deemed as a critical reference when considering the modification strategy. Guided by the information of structural biology, some advances in the chemical modification of aminoglycosides have been achieved [13,14,15,16,17]. Herein, we report a modifying strategy for kanamycin based on the structural information.

## 2. Result and Discussion

### 2.1. Design

**Figure 1:**
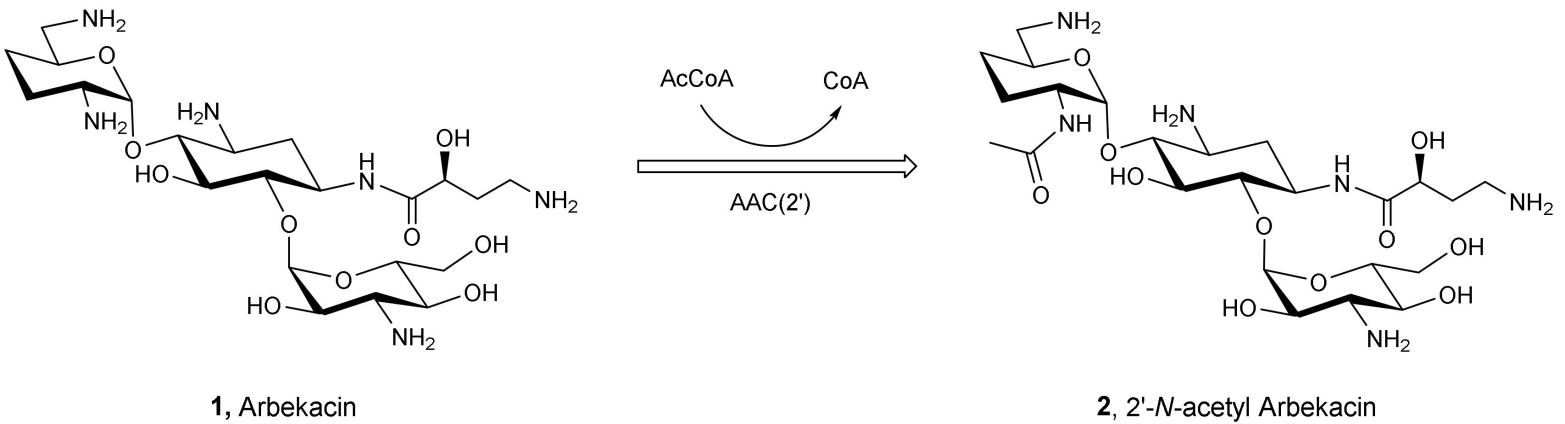
Schematic illustration for the action of AAC(2′) on arbekacin.

**Figure 2:**
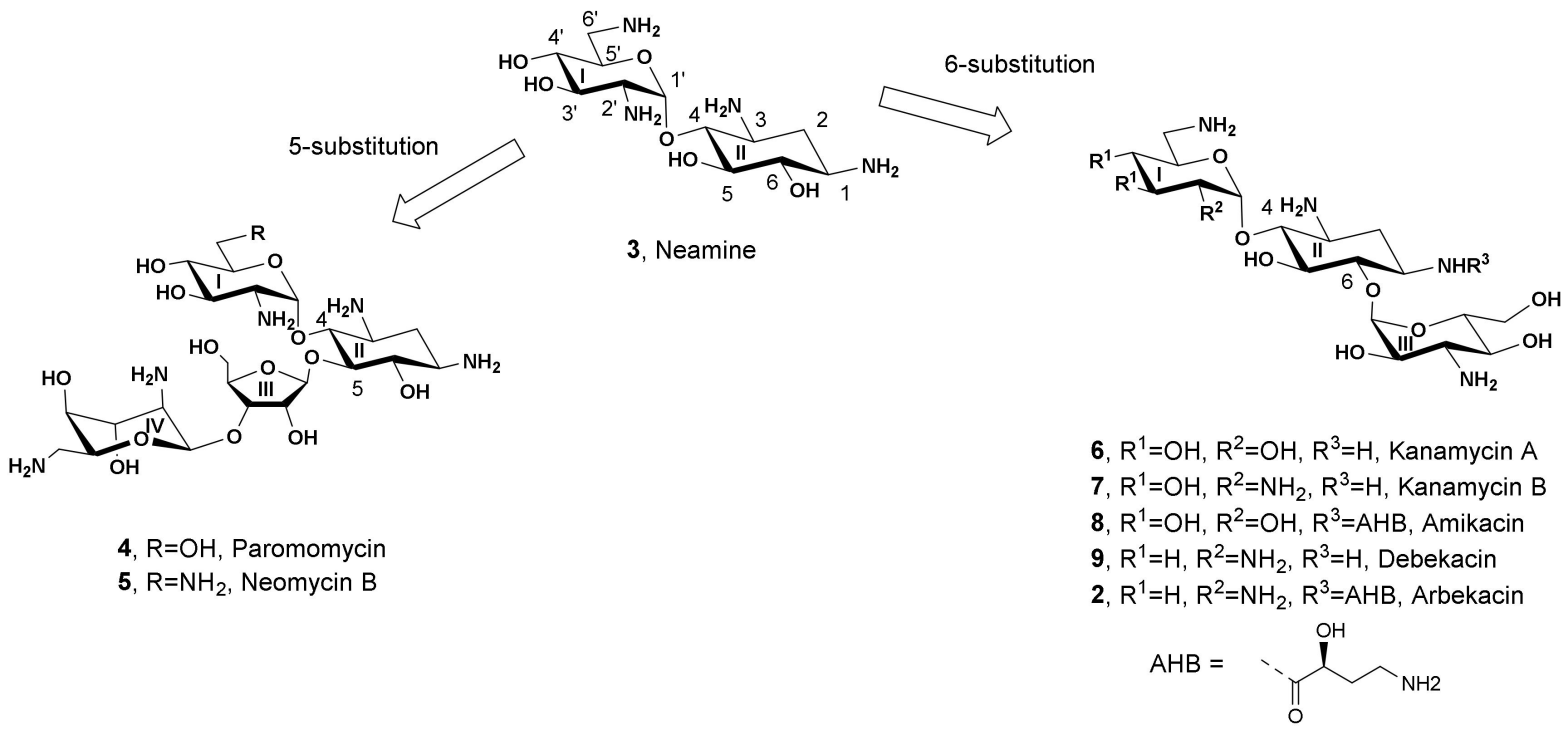
Structures of neamine and several representative aminoglycosides. The neamine core is composed of ring I and ring II.

**Figure 3:**
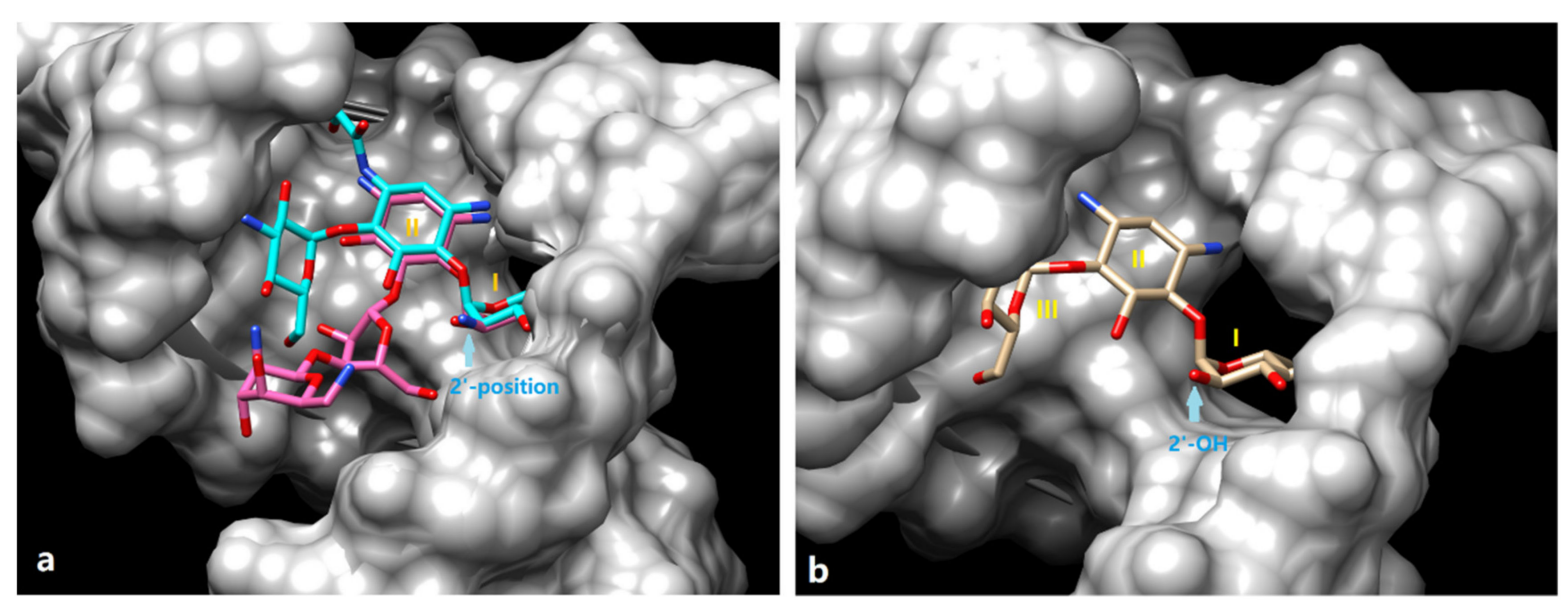
Crystal structures of neamine-containing aminoglycosides binding to A site. (**a**) The complexes of amikacin (cyanic, PDB ID: 4P20) and neomycin B (pink, PDB ID: 2ET4) with the decoding site are superimposed (based on nucleic acid chains). The neamine moiety is marked with Roman numerals I and II. The nucleic acid chains are colored in grey; (**b**) the complex of kanamycin A binding to A site (PDB ID: 2ESI). In both figures, 2′-position is indicated.

It is well known that aminoglycoside-modifying enzymes include three categories: aminoglycoside phosphotransferases (APHs), aminoglycoside acetyltransferases (AACs), and aminoglycoside nucleotidyltransferase (ANTs). Among them, AACs can add acetyl groups to the corresponding free amino groups on aminoglycosides in the presence of acetyl coenzyme A. The acylated products usually lose antibacterial activity. However, in a research paper on AAC (2′), it was noted that some 2′-*N*-acetylated products definitely remained active; although they were much weaker than their parent compounds [18]. For example, 2′-*N*-acetyl arbekacin (**2**) showed a considerably low MIC (minimum inhibitory concentration) range of 1.56–3.13 µg/mL against a variety of bacteria, while its parent compound arbekacin (**1**) showed a range of 0.20–0.78 µg/mL (Figure 1). For another example, the 2′-*N*-acetyl neomycin also showed distinct activity in the same research. These facts suggest that modification on 2′-amino does not necessarily mean completely losing activity. Since arbekacin and neomycin share the same core structure, namely neamine (**3**), a reasonable inference is that such a modification does not block the binding of the drugs to their target profoundly. In recent years, some 3D structures of A Site in complex with aminoglycosides have been disclosed, which affords a brand-new perspective to explain such a phenomenon. The common aminoglycosides usually contain a pseudodisaccharide unit named neamine (or its variants), and the extra substituent attaches on the 5-position or 6-position of 2-deoxystreptamine (2-DOS, ring II) (Figure 2). Despite the distinct structural difference, the 3D complexes show that the neamine moiety still binds to A site in a very similar way, whether in terms of the binding position or the binding conformation (Figure 3a) [19,20]. Another important feature is that the binding site looks quite spacious, and the 2′-NH_2_ (or 2′-OH) points to a vacant space of the major groove of the RNA helix. Under this condition, an additional acetyl could be well accommodated, and then 2′-*N*-acetylated aminoglycosides could still bind to A Site and retain a part of activity. Since the 2′-position is closely adjacent to the acidic backbone of the nucleic acid, acylation of the amino group on this site may significantly reduce the electrostatic force between them, which may account for the partial loss of activity. In studies with AAC(2′)s, some chemical modification on C-2′ had been carried out [21]. Neomycin, paromomycin, and ribostamycin had been used as targets for modification, and several small groups, such as methyl, ethyl, propyl, or glycyl were installed on 2′-NH_2_. Some modified products showed good activity against AAC(2′)-producing bacteria. 

Usually, AAC(2′)s do not modify 2′-OH, so the antibiotics with a hydroxyl group on C-2′, such as kanamycin A, can remain active on the bacteria expressing AAC(2′)s [18]. However, 2′-OH could be a potential target for modifying enzyme APHs and/or ANTs, which can attach a phosphoryl or a nucleotidyl to a hydroxyl group, and then lead to resistance, so a proactive chemical modification seems meaningful. On the other hand, since the room that 2′-substituents point to is so vacant, a variety of groups with different features should also be able to be integrated into this position (Figure 3b). Based on these considerations, we used kanamycin A as parent compound, designed, synthesized a series of 2′-modified derivatives, and evaluated, and the relationship between activity and the features of introduced groups was also assayed. 

### 2.2. Synthesis

**Scheme 1:**
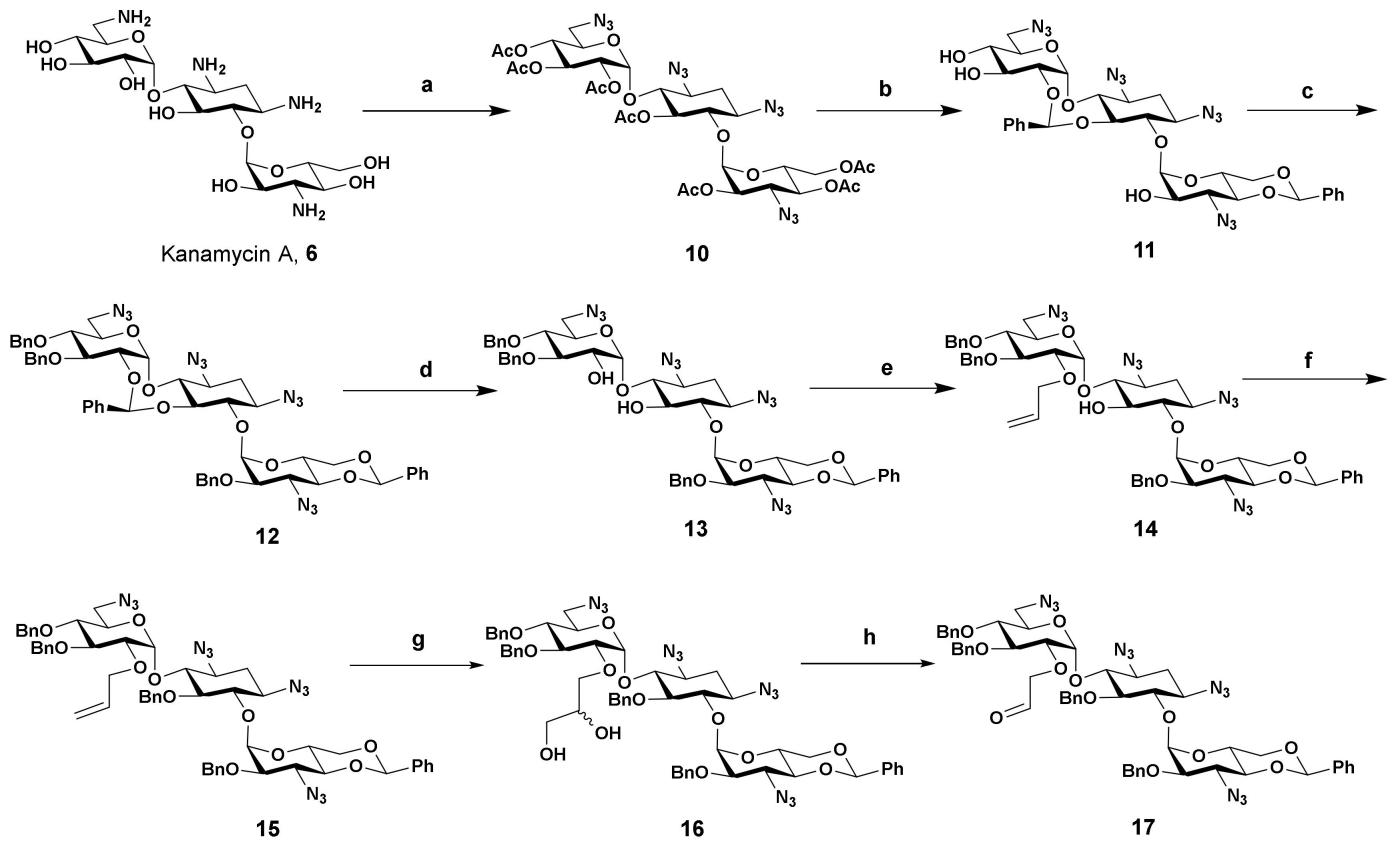
Conditions: (**a**) (**i**) TfN_3_, CuSO_4_, CH_3_CN, H_2_O, NEt_3_, ice bath; (**ii**) Ac_2_O, pyridine, rt, 86% over two steps; (**b**) (**i**) MeOH, NaOMe, rt, 99%; (**ii**) PhCH(OCH_3_)_2_, DMF, p-TsOH, 68 °C, 46%; (**c**) BnBr, NaH, DMF, 93%; (**d**) (**i**)THF, MeOH, HCl (aq), 50 °C, 93%; (**ii**) 1.2 equiv of PhCH(OCH_3_)_2_, CH_3_CN, p-TsOH, rt, 75%; (**e**) Allyl bromide, Ag_2_O, TBAI, Toluene, 90%; (**f**) BnBr, NaH, DMF, 86%; (**g**) K_2_OsO_4_•2H_2_O, NMO, H_2_O, acetone, 57%; (**h**) NaIO_4_, MeOH, CH_2_Cl_2_, 100%.

**Scheme 2:**
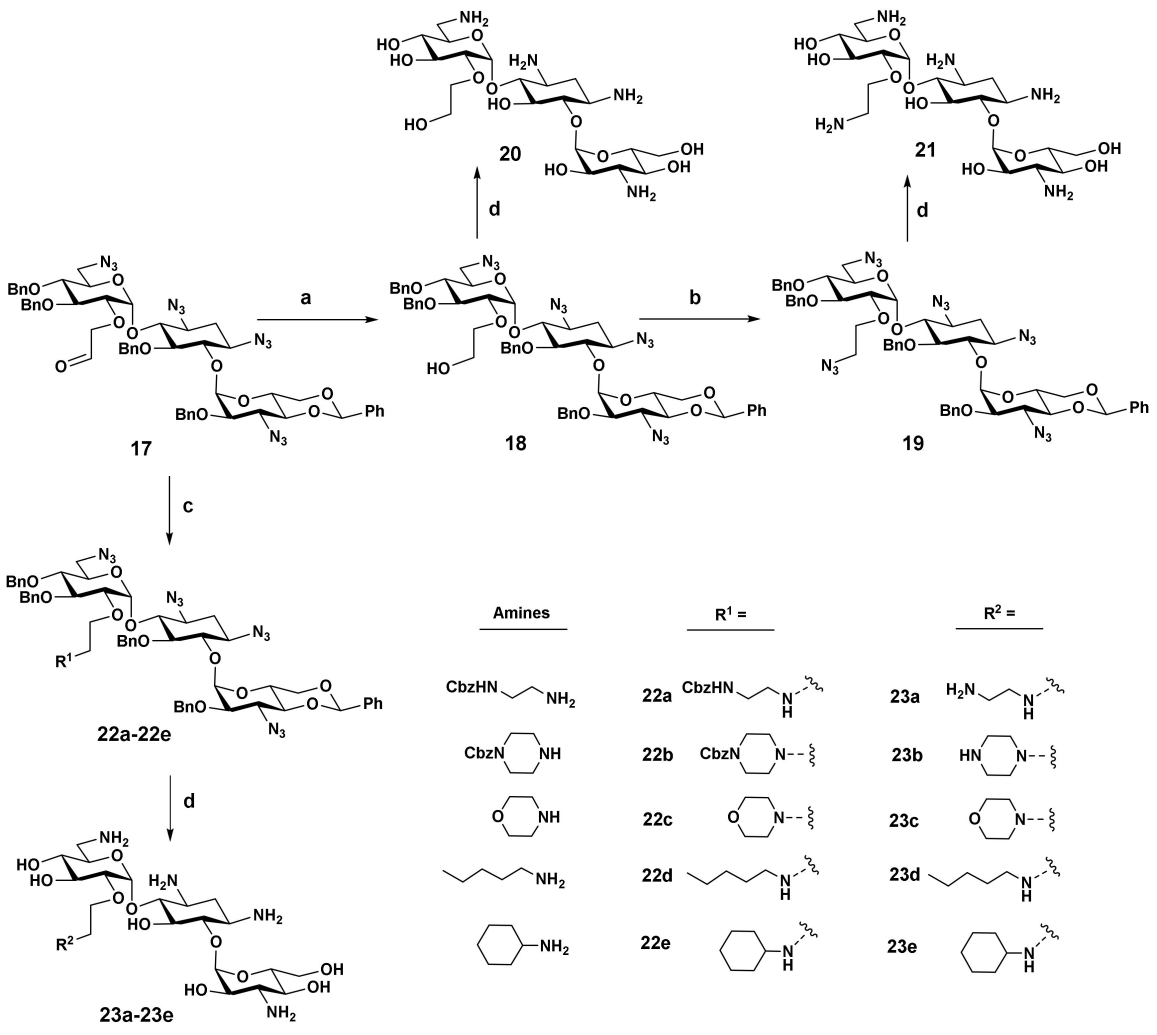
Conditions: (**a**) NaBH_4_, MeOH, CH_2_Cl_2_, ice bath, 92%; (**b**) (**i**) TsCl, Pyridine, rt; (**ii**) NaN_3_, DMF, 84% over two steps; (**c**) Amines, NaBH(OAc)_3_, 1,2-dichloroethane, rt, 52–67%; (**d**) (**i**) PMe_3_, THF, H_2_O, NaOH; (**ii**) H_2_, Pd/C, MeOH, THF, 55–78% over two steps.

Our synthesis started with commercially available sulfate of kanamycin A (**6**) (Scheme 1). According to the literature, we firstly synthesized 1,3,6′,3″-tetraazido-4″,6″-*O*-benzylidene-3′,4′,2″-tri-*O*-benzylkanamycin A (**13**) through seven steps [22]. Following this, after a silver oxide-promoted regioselective allylation, we successfully obtained key 2′-allylated kanamycin A (**14**), which was confirmed by a serial of 2D NMR spectroscopy. Benzylation of 5-OH of **14** gave fully protected intermediate **15**, and then a potassium osmate catalyzed dihydroxylation in the presence of *N*-methylmorpholine-*N*-oxide was carried out; **15** smoothly transformed into diol **16** in high yield. Following this, treatment of the resulting diol with sodium periodate afforded key intermediate aldehyde **17**. Reduction of **17** with sodium borohydride gave alcohol **18** (Scheme 2). After this, tosylation and then substitution with sodium azide achieved **19**. Compound **18** and **19** underwent a Staudinger reduction and then a palladium-catalyzed hydrogenation, and finally transformed into products **20** and **21**, respectively. On the other hand, **17** coupled with different amines via reductive amination to afford **22a**–**22e**. All these intermediates transformed into the final products **23a**–**23e** with the same procedure as for **18** and **19**.

### 2.3. Bioactivity Assay

In order to determine the activity of newly synthesized compounds, we chose ten typical bacteria as test objects, both Gram-positive and Gram-negative bacteria were included. It contained two strains of *E. coli* (ATCC 25922 and ATCC 35218, G^−^), three strains of *S. aureus* (ATCC 29213, ATCC 25923, and ATCC 33591, G^+^), two strains of *K. pneumoniae* (ATCC 70063 and ATCC 13883, G^−^), one strain of *E. faecalis* (ATCC 29212, G^+^), and two strains of *P. aeruginosa* (ATCC 27853 and PAO1, G^−^). All compounds were tested against these microorganisms by the microdilution assay, and the minimum inhibitory concentrations (MIC) in µg/mL were recorded. Kanamycin A served as the control.

As shown in Table 1, most compounds exhibited distinct activity, and the activity was close to their parent compound kanamycin A generally. Among them, compound **21** showed somewhat better potency than kanamycin A. Except in the case of ATCC 25922, the MIC values of compound **21** were lower than or equal to that of kanamycin A in the case of the other nine bacteria. When it comes to compound **20**, in comparison with kanamycin A, the activity did not increase. The major structural difference between kanamycin A, compound **20**, and **21** is that compound **21** has one more amino group than the other two. It looks like that additional amino groups may benefit the activity. However, compound **23a**, which is the product obtained by adding a second amino group at the end of the 2′-position of compound **20**, did not exhibit further improvement in activity. On the contrary, the activity was slightly reduced in several cases in comparison with compound **21**. In the case of compound **23b**, in which the flexible 2-amino-ethylamino of **23a** was replaced by a more rigid piperazinyl, the activity had also not improved. When the piperazinyl in compound **23b** was replaced by a similarly hydrophilic morpholine ring, the resulting product compound, **23c**, gave almost the same activity result as **23b**. After studying the impact of the terminal hydrophilic groups on activity, we also checked whether the hydrophobic groups on the end of the 2′-position affected the activity. Compound **23d** and **23e** bear a pentyl and a cyclohexyl, respectively, at the terminal of 2′-substituent. To our surprise, these two compounds displayed comparable activity to that of other new derivatives. Compound **23e**, which bears a bulky and rigid cyclohexyl, even showed a very similar result to that of compound **21**. Based on these results, we can conclude that hydrophobicity and basicity of the terminal of 2′-substituents do not affect the activity remarkably, and the size of the groups also does not. Since the space that 2′-postion faces is so spacious and empty, it is no surprise that a good tolerance for the diversity of the introduced groups can be observed during chemical modifying.

Among the tested bacteria, some are antibiotic-resistant strains. *K. pneumoniae* K6 (ATCC 700603) is a clinical isolate and is reported to be resistant to some aminoglycosides (caused by ANT(2″)). Our antimicrobial susceptibility test showed that it was resistant to kanamycin A (MIC = 16 µg/mL). The bioassay results showed our modifying strategy brought some advantage, and all new compounds gave lower or equal MIC values to kanamycin A. Of note, **21**, **23a**, and **23f** displayed a four- to eight-fold enhancement in activity. ATCC 33591 is a methicillin-resistant *S. aureus* (MRSA). Many MRSA strains contain genes encoded for APH(3′), ANT(4′), and AAC(6′)/APH(2″), which render the bacteria resistant to many aminoglycosides. In our research, the MIC for kanamycin A against ATCC 33591 is 64 µg/mL. Three compounds, **21**, **23a**, and **23e** displayed eight-times enhancement in activity. In the case of PAO1, a prominent pathogen for its intrinsic resistance to many antibiotics, among newly synthesized derivatives, **21** also showed four-fold more potency than kanamycin. However, in the cases of the drug-resistant ATCC29212 (expressing efflux pump) and ATCC 27853 (expressing APH(3′)), none of new compounds exhibited a better outcome. In general, modification on the 2′-position may bring some benefits in treating drug-resistant bacteria, but this kind of improvement is mild and hard to predict.

## 3. Materials and Methods 

### 3.1. Chemistry

General: All commercial reagents were used without further purification unless otherwise stated. Anhydrous *N*,*N*-dimethylformamide (DMF) was obtained by distilling commercial product over P_2_O_5_ under reduced pressure. Routine ^1^H and ^13^C nuclear magnetic resonance spectra were recorded on the Bruker AVANCE III 400 (Bruker Scientific Co. Ltd., Zurich, Switzerland) spectrometer or Bruker AVANCE III 600 spectrometer (Bruker Scientific Co. Ltd., Zurich, Switzerland). Samples were dissolved in deuterated chloroform (CDCl_3_) or deuterium oxide (D_2_O), and tetramethylsilane (TMS) was used as reference. High resolution mass spectra were recorded on the Thermo Scientific Orbitrap Fusion Lumos Mass Spectrometer (Thermo Fisher Scientific Inc., San Jose, CA, USA). Analytical thin-layer chromatography (TLC) was performed on Merck Silica Gel 60 F254. Compounds were visualized by UV light (254 nm) and/or by staining with a yellow solution containing Ce(NH_4_)_2_(NO_3_)_6_ (0.5 g) and (NH_4_)_6_Mo_7_O_24_•4H_2_O (24.0 g) in 6% H_2_SO_4_ (500 mL) or ninhydrin solution in ethyl acetate (5%) followed by heating. NMR spectra for new compounds can be found in Supplementary Materials section.

*2′-O-Allyl-1,3,6′,3″-tetraazido-4″,6″-O-benzylidene-3′,4′,2″-tri-O-benzylkanamycin A* (**14**): To a flask was added **13** (121 mg, 0.13 mmol), toluene (2 mL), freshly prepared silver (I) oxide (80 mg, 0.34 mmol), tetrabutylammonium iodide (10 mg, 0.03 mmol) and the allyl bromide (35mg, 0.29 mmol). The mixture was stirred overnight at room temperature and TLC showed the starting material disappeared. The reaction mixture was filtered and the filtrate was concentrated to residue. The gross product was purified by silica gel chromatography with the mixed solvent (petroleum/ethyl acetate from 15:1 to 6:1) as eluent to give the titled compound (112 mg, 0.12 mmol, 90% yield) as a colorless semisolid. ^1^H NMR (600 MHz, CDCl_3_) δ 7.50–7.49 (m, 2H), 7.43–7.42 (m, 2H), 7.39–7.26 (m, 16H), 5.91–5.85 (m, 1H), 5.52 (s, 1H), 5.25–5.22 (m, 2H), 5.18–5.16 (m, 1H), 5.09 (d, *J* = 3.6 Hz, 1H), 4.91–4.83 (m, 4H), 4.77 (d, *J* = 11.8 Hz, 1H), 4.70 (d, *J* = 1.7 Hz, 1H), 4.62 (d, *J* = 11.0 Hz, 1H), 4.48 (td, *J*_1_ = 5.0 Hz, *J*_2_ = 10.0 Hz, 1H), 4.34–4.31 (m, 1H), 4.22 (dd, *J*_1_ = 5.0 Hz, *J*_2_ = 10.0 Hz, 1H), 4.18–4.15 (m, 1H), 4.07–4.02 (m, 2H), 3.96 (t, *J* = 9.4 Hz, 1H), 3.65–3.54 (m, 5H), 3.50 (d, *J*_1_ = 3.6 Hz, *J*2 = 9.8 Hz, 1H), 3.45–3.63 (m, 4H), 3.30–3.21 (m, 2H), 2.43 (ddd, *J*_1_ = *J*_2_ = 4.5 Hz, *J*_3_ = 13.2 Hz, 1H), 1.60 (ddd, *J*_1_ = *J_2_* = *J*_3_ = 12.6 Hz, 1H). ^13^C NMR (150 MHz, CDCl_3_) δ 138.17, 137.77, 137.18, 137.09, 133.14, 128.95, 128.54, 128.48, 128.22, 128.21, 128.13, 128.01, 127.90, 127.80, 127.78, 126.04, 119.43, 101.32, 101.20, 97.64, 86.51, 81.81, 80.20, 80.09, 79.79, 78.10, 77.54, 75.60, 75.31, 74.13, 74.08, 73.10, 71.09, 68.78, 62.78, 61.94, 60.38, 58.76, 51.19, 32.21. HRMS (ESI/APCI) calculated for (C_49_H_54_N_12_O_11_Na) [M + Na^+^]: 1009.3927, found: 1009.3962.
*2′-O-Allyl-1,3,6′,3″-tetraazido-4″,6″-O-benzylidene-5,3′,4′,2″-tetra-O-benzylkanamycin A* (**15**): To a stirred solution of **14** (226 mg, 0.23 mmol) in anhydrous DMF (3 mL) was added sodium hydride (26 mg, 60% in mineral oil, 0.65 mmol) and the resulting mixture was stirred for 30 min at room temperature, then benzyl bromide (59 mg, 0.35 mmol) was added in one portion. After stirring for 4 h, the reaction mixture was poured into water (50 mL) and the aqueous layer was extracted with ethyl acetate (3 × 15 mL). The combined organic phase was washed with brine, dried over anhydrous sodium sulfate, and concentrated under a vacuum. The residue was purified by silica gel column chromatography with the mixed solvent (petroleum ether/ethyl acetate 15:1 to 9:1) as eluent to give product **15** (217 mg, 0.20 mmol, 86% yield) as a white solid. ^1^H NMR (400 MHz, CDCl_3_) δ 7.44–7.24 (m, 20H), 7.19–7.11 (m, 4H), 7.05 (t, *J* = 7.4 Hz, 1H), 5.55 (d, *J* = 3.7 Hz, 1H), 5.52 (d, *J* = 4.0 Hz, 1H), 5.51–5.43 (m, 1H), 5.28 (s, 1H), 5.10 (d, *J* = 12.4 Hz, 1H), 4.98–4.74 (m, 8H), 4.58 (d, *J* = 11.3 Hz, 1H), 4.31–4.27 (m, 1H), 4.02–3.92 (m, 3H), 3.86–3.80 (m, 2H), 3.75 (d, *J* = 9.4 Hz, 1H), 3.67–3.50 (m, 6H), 3.46–3.36 (m, 4H), 3.28 (dd, *J*_1_ = 3.8 Hz, *J*2 = 9.9 Hz, 1H), 3.22 (t, *J* = 9.8 Hz, 1H), 2.40 (ddd, *J*_1_ = *J*_2_ = 4.5 Hz, *J*3 = 13.2 Hz, 1H), 1.54 (ddd, *J*_1_ = *J*_2_ = *J*_3_ = 12.9 Hz, 1H). ^13^C NMR (100 MHz, CDCl3) δ 138.45, 138.04, 137.58, 137.27, 136.78, 134.25, 128.84, 128.59, 128.46, 128.44, 128.37, 128.19, 128.17, 127.92, 127.83, 127.74, 127.62, 127.10, 126.32, 125.19, 117.48, 101.39, 97.33, 96.32, 82.83, 81.81, 79.59, 78.70, 78.28, 78.11, 77.92, 77.15, 75.52, 74.96, 74.51, 73.44, 73.31, 70.76, 68.58, 62.89, 61.47, 60.17, 59.19, 51.37, 32.16. HRMS (ESI/APCI) calculated for (C_56_H_60_N_12_O_11_Na) [M + Na^+^]: 1099.4397, found: 1099.4412.
*2′-O-(2,3-Dihydroxypropyl)-1,3,6′,3″-tetraazido-4″,6″-O-benzylidene-5,3′,4′,2″-tetra-O-benzylkanamycin A* (**16**): To a flask was added **15** (213 mg, 0.20 mmol), potassium osmate (VI) dihydrate (4 mg, 0.01 mmol), *N*-methylmorpholine-*N*-oxide solution in water (94 mg, 0.40 mmol, 50% *w*/*w*), acetone (5 mL), and water (0.1 mL) in sequence. After stirring overnight at room temperature, TLC showed the reaction was completed. The reaction mixture was poured into aqueous solution of sodium thiosulfate pentahydrate (50 mL, 1% *w*/*w*) and stirred for 30 min, then the resulting mixture was extracted with ethyl acetate (3 × 10 mL). The organic layer was combined, dried over anhydrous sodium sulfate, and concentrated under a vacuum. The gross product was purified by silica gel chromatography with the mixed solvent (petroleum/ethyl acetate from 8:1 to 4:1) as the solvent to give the titled compound **16** (125 mg, 0.11 mmol, 57% yield) as a colorless semisolid. ^1^H NMR (400 MHz, CDCl_3_) δ 7.44–7.14 (m, 24H), 7.10 (t, *J* = 7.2 Hz, 1H), 5.54 (t, *J* = 4.0 Hz, 1H), 5.48 (t, *J* = 4.1 Hz, 1H), 5.34 (s, 1H), 5.03–4.94 (m, 2H), 4.86 (d, *J* = 11.2 Hz, 1H), 4.82–4.68 (m, 4H), 4.59 (d, *J* = 11.2 Hz, 1H), 4.21–4.08 (m, 2H), 3.94–3.87 (m, 2H), 3.79–3.42 (m, 9H), 3.41–3.15 (m, 8H), 3.08 (br, 1H), 2.43–2.35 (m, 1H), 1.68–1.58 (m, 1H). HRMS (ESI/APCI) calculated for (C_56_H_62_N_12_NaO_13_) [M + Na^+^]: 1133.4452, found: 1133.4487.
*2′-O-(2-Oxoethyl)-1,3,6′,3″-tetraazido-4″,6″-O-benzylidene-5,3′,4′,2″-tetra-O-benzylkanamycin A* (**17**): To a flask was added **16** (125 mg, 0.11 mmol), methanol (5 mL), and sodium periodate (47 mg, 0.22 mmol) and the resulting mixture was stirred at room temperature. After 4 h, TLC showed all starting material was consumed. The reaction mixture was poured into aqueous solution of sodium thiosulfate pentahydrate (50 mL, 0.4% *w*/*w*) and stirred for 30 min, then the resulting mixture was extracted with ethyl acetate (3 × 10 mL). The organic layer was combined, dried over anhydrous sodium sulfate, and concentrated under a vacuum. The resulting product **17** (122 mg, 0.11 mmol, 100%) was used in the following reactions directly without further purification. ^1^H NMR (400 MHz, CDCl_3_) δ ^1^H NMR (400 MHz, CDCl_3_) δ 9.09 (s, 1H), 7.44–7.16 (m, 22H), 7.10–7.04 (m, 3H), 5.56 (d, *J* = 3.8 Hz, 1H), 5.51 (d, *J* = 3.8 Hz, 1H), 5.28 (s, 1H), 5.07 (d, *J* = 12.5 Hz, 2H), 4.96 (d, *J* = 12.4 Hz, 1H), 4.86-4.76 (m, 4H), 4.68 (d, *J* = 11.2 Hz, 1H), 4.59 (d, *J* = 11.2 Hz, 1H), 4.29–4.25 (m, 1H), 4.03–3.96 (m, 3H), 3.86–3.79 (m, 2H), 3.74–3.61 (m, 3H), 3.59–3.49 (m, 3H), 3.46–3.38 (m, 5H), 3.25–3.19 (m, 2H), 2.41 (ddd, *J*_1_ = *J*_2_ = 4.7 Hz, *J*_3_ = 13.0 Hz, 1H), 1.68 (ddd, *J*_1_ = *J*_2_ = *J*_3_ = 12.6 Hz, 1H). HRMS (ESI/APCI) calculated for (C_55_H_58_N_12_NaO_12_) [M + Na^+^]: 1101.4189, found: 1101.4218.
*2′-O-(2-Hydroxyethyl)-1,3,6′,3″-tetraazido-4″,6″-O-benzylidene-5,3′,4′,2″-tetra-O-benzylkanamycin A* (**18**): The product **17** (110 mg, 0.10 mmol) was dissolved in the mixture of methanol (3 mL) and dichloromethane (2 mL), and to the solution was added sodium borohydride (15 mg, 0.40 mmol) in several portions while stirring at ice bath temperature. After 30 min, TLC showed the reaction was completed. The solvent was removed, and the gross product was purified with silica gel chromatography by using the mixed solvent (petroleum ether/ethyl acetate 20:1 to 10:1) as eluent to give product **18** (101 mg, 0.09 mmol, 92% yield) as a white semisolid. ^1^H NMR (400 MHz, CDCl_3_) δ 7.43–7.24 (m, 20H), 7.20–7.15 (m, 4H), 7.08 (t, *J* = 7.2 Hz, 1H), 5.53 (d, *J* = 4.2 Hz, 1H), 5.52 (d, *J* = 4.3 Hz, 1H), 5.31 (s, 1H), 5.05 (d, *J* = 12.3 Hz, 1H), 4.97 (d, *J* = 12.2 Hz, 1H), 4.88 (d, *J* = 11.2 Hz, 1H), 4.82–4.75 (m, 4H), 4.59 (d, *J* = 11.3 Hz, 1H), 4.25–4.21 (m, 1H), 4.06 (dd, *J*_1_ = 5.0 Hz, *J*_2_ = 10.2 Hz, 1H), 3.96-3.85 (m, 2H), 3.66–3.29 (m, 14 H), 3.24 (t, *J* = 9.8 Hz, 1H), 2.65 (t, *J* = 6.1 Hz, 1H), 2.39 (dt, *J*_1_ = 4.3 Hz, *J*_2_ = 13.2, Hz, 1H), 1.63 (ddd, *J*_1_ = *J*_2_ = *J*_3_ = 12.7 Hz, 1H). ^13C NMR (100 MHz, CDCl^_3_) δ 137.97, 137.83, 137.26, 137.23, 136.81, 128.87, 128.63, 128.58, 128.50, 128.47, 128.18, 128.16, 127.97, 127.93, 127.83, 127.76, 127.47, 126.27, 125.72, 101.41, 97.21, 96.43, 82.29, 81.35, 80.54, 79.60, 78.55, 78.43, 78.03, 77.76, 75.80, 75.03, 74.95, 73.45, 73.29, 71.01, 68.60, 62.91, 62.31, 61.40, 60.18, 59.92, 51.20, 32.35. HRMS (ESI/APCI) calculated for (C_55_H_60_N_12_NaO_12_) [M + Na^+^]: 1103.4346, found: 1103.4369.
*2′-O-(2-Azidoethyl)-1,3,6′,3″-tetraazido-4″,6″-O-benzylidene-5,3′,4′,2″-tetra-O-benzylkanamycin A* (**19**): To the solution of **18** (65 mg, 0.06 mmol) in pyridine (3 mL) was added p-toluene sulfonyl chloride (46 mg, 0.18 mmol) in one portion, and the resulting mixture was stirred overnight. Then, the solvent was removed, and the residue was purified by column chromatography on silica gel using petroleum ether/ethyl acetate (10:1 to 5:1) as eluent to afford the tosylate intermediate, which was mixed with sodium azide (12 mg, 0.18 mmol) and DMF (3 mL). After stirring at 80 °C for 4 h, the reaction mixture was poured into 50 mL of water and the aqueous layer was extracted with ethyl acetate (3 × 10 mL). The combined organic phase was dried over Na_2_SO_4_ and concentrated under a vacuum. The crude was purified by column chromatography on silica gel by using the mixture of petroleum ether and ethyl acetate (10:1 to 5:1) as eluent to afford **19** (56 mg, 0.05 mmol, 84% yield). ^1^H NMR (600 MHz, CDCl_3_) δ 7.43–7.38 (m, 4H), 7.35–7.24 (m, 16H), 7.18 (t, *J* = 7.7 Hz, 2H), 7.12–7.06 (m, 3H), 5.56–5.55 (m, 2H), 5.29 (s, 1H), 5.09 (d, *J* = 12.3 Hz, 1H), 4.99 (d, *J* = 12.3 Hz, 1H), 4.87–4.76 (m, 5H), 4.58 (d, *J* = 11.3 Hz, 1H), 4.29–4.26 (m, 1H), 4.01 (dd, *J*_1_ = 4.9 Hz, *J*_2_ = 10.1 Hz, 1H), 3.95 (t, *J* = 9.4 Hz, 1H), 3.87–3.82 (m, 2H), 3.75 (t, *J* = 9.4 Hz, 1H), 3.66 (t, *J* = 9.4 Hz, 1H), 3.61–3.51 (m, 5H), 3.46–3.36 (m, 4H), 3.25–3.22 (m, 2H), 3.15–3.11 (m, 1H), 3.04–2.96 (m, 2H), 2.40 (ddd, *J*_1_ = *J*_2_ = 4.6 Hz, *J*_3_ = 13.3, Hz, 1H), 1.64 (ddd, *J*_1_ = *J*_2_ = *J*_3_ = 12.7 Hz, 1H). ^13^C NMR (150 MHz, CDCl_3_) δ 138.39, 137.90, 137.54, 137.21, 136.74, 128.85, 128.58, 128.45, 128.41, 128.19, 127.92, 127.87, 127.82, 127.63, 127.60, 127.22, 126.29, 125.14, 101.39, 96.89, 96.32, 82.78, 81.58, 80.20, 79.55, 78.35, 78.01, 77.76, 75.41, 74.94, 74.46, 73.30, 70.76, 70.75, 68.57, 62.87, 61.46, 60.15, 59.25, 51.25, 50.81, 32.14. HRMS (ESI/APCI) calculated for (C_55_H_59_N_15_NaO_11_) [M + Na^+^]: 1128.4411, found: 1128.4442.
*2′-O-[[2-[2-(benzyloxycarbonylamino)ethyl]amino]-ethyl]-1,3,6′,3″-tetraazido-4″,6″-O-benzylidene-5,3′,4′,2″-tetra-O-benzylkanamycin A* (**22a**): To a flask was added **17** (53 mg, 0.05 mmol), 1,2-dichloroethane (3 mL), and 1-(benzyloxycarbonylamino)-2-aminoethane (48 mg, 0.25 mmol) in one portion. The resulting mixture was stirred for 30 min, then sodium triacetoxyborohyride (53 mg, 0.25 mmol) was added in portions. After stirring overnight at room temperature, TLC showed a minor amount of **17** remained. Then, another portion of sodium triacetoxyborohyride (32 mg, 0.15 mmol) was added. After stirring for another 8 h, the reaction was complete. The solvent was removed, and the residue was purified by silica gel chromatography (petroleum/ethyl acetate from 8:1 to 4:1) to give the titled compound **22a** (38 mg, 0.03 mmol, 67% yield) as a colorless semisolid. ^1^H NMR (400 MHz, CDCl_3_) δ 7.45–7.09 (m, 29H), 7.06 (t, *J* = 7.2 Hz, 1H), 5.57 (d, *J* = 3.6 Hz, 1H), 5.53 (d, *J* = 3.6 Hz, 1H), 5.33 (s, 1H), 5.06 (s, 2H), 5.01–4.93 (m, 2H), 4.88–4.74 (m, 5H), 4.58 (d, *J* = 11.2 Hz, 1H), 4.26–4.20 (m, 1H), 4.02 (dd, *J*_1_ = 10.1, *J*_2_ = 4.8 Hz, 1H), 3.96–3.80 (m, 3H), 3.75 (t, *J* = 9.4 Hz, 1H), 3.68 (t, *J* = 9.4 Hz, 1H), 3.62–3.21 (m, 13H), 3.01 (br, 2H), 2.53–2.28 (m, 5H), 1.65 (ddd, *J*_1_ = *J*_2_ = *J*_3_ = 12.6 Hz, 1H). ^13^C NMR (100 MHz, CDCl_3_) δ 156.46, 138.30, 137.83, 137.28, 136.78, 136.64, 128.88, 128.58, 128.49, 128.18, 128.15, 128.10, 128.06, 127.98, 127.93, 127.89, 127.76, 127.53, 127.41, 126.23, 125.96, 101.35, 96.77, 96.24, 82.64, 81.23, 80.15, 79.66, 78.46, 78.15, 78.05, 77.70, 75.38, 74.96, 74.90, 73.30, 70.93, 68.61, 66.60, 62.94, 61.47, 60.09, 59.71, 51.29, 48.60, 48.34, 39.80, 32.19. HRMS (ESI/APCI) calculated for (C_65_H_73_N_14_O_13_) [M + H^+^]: 1257.5476, found: 1257.5442.
*2′-O-[2-(4-benzyloxycarbonylpiperizyl)-ethyl]-1,3,6′,3″-tetraazido-4″,6″-O-benzylidene-5,3′,4′,2″-tetra-O-benzylkanamycin A* (**22b**): This compound was synthesized through reductive amination by coupling **17** with 1-benzyloxycarbonylpiperize with the same procedure as described in the preparation of **22a**. 55% yield, colorless semisolid. ^1^H NMR (400 MHz, CDCl_3_) δ 7.43–7.22 (m, 25H), 7.16–7.10 (m, 4H), 7.00 (t, *J* = 7.5 Hz, 1H), 5.56 (d, *J* = 3.6 Hz, 1H), 5.54 (d, *J* = 3.7 Hz, 1H), 5.28 (s, 1H), 5.10–5.06 (m, 3H), 4.93 (d, *J* = 12.1 Hz, 1H), 4.86–4.75 (m, 5H), 4.58 (d, *J* = 11.2 Hz, 1H), 4.31–4.27 (m, 1H), 3.97 (dd, *J*_1_ = 5.0 Hz, *J*_2_ = 10.3 Hz, 1H), 3.91 (t, *J* = 9.4 Hz, 1H), 3.84–3.72 (m, 3H), 3.67–3.36 (m, 10H), 3.30–3.20 (m, 7H), 2.40 (ddd, *J*_1_ = *J*_2_ = 4.4 Hz, *J*3 = 13.1Hz, 1H), 2.29–2.22 (m, 1H), 2.12–1.94 (m, 5H), 1.64 (ddd, *J*_1_ = *J*_2_ = *J*_3_ = 12.7 Hz, 1H). ^13^C NMR (100 MHz, CDCl_3_) δ 155.09, 138.45, 137.84, 137.46, 137.21, 136.73, 128.85, 128.59, 128.52, 128.47, 128.39, 128.18, 128.09, 127.92, 127.56, 127.29, 127.16, 126.25, 125.24, 101.25, 97.18, 96.28, 82.67, 81.52, 80.11, 79.55, 78.3, 77.94, 77.71, 75.16, 74.95, 73.3, 70.8, 68.52, 67.12, 62.83, 61.43, 60.12, 59.22, 57.30, 52.88, 51.25, 43.46, 32.16, 29.70. HRMS (ESI/APCI) calculated for (C_67_H_75_N_14_O_13_) [M + H^+^]: 1283.5633, found: 1283.5602.
*2′-O-[2-(4-Morpholinyl)-ethyl]-1,3,6′,3″-tetraazido-4″,6″-O-benzylidene-5,3′,4′,2″-tetra-O-benzylkanamycin A* (**22c**): This compound was synthesized through reductive amination by coupling **17** with morpholine with the same procedure as described in the preparation of **22a**. 68% yield, colorless semisolid. ^1^H NMR (400 MHz, CDCl_3_) δ 7.44–7.21 (m, 20H), 7.17–7.10 (m, 4H), 7.01 (t, *J* = 7.4 Hz, 1H), 5.57 (d, *J* = 3.7 Hz, 1H), 5.54 (d, *J* = 3.7 Hz, 1H), 5.28 (s, 1H), 5.08 (d, *J* = 12.2 Hz, 1H), 4.93 (d, *J* = 12.2 Hz, 1H), 4.89–4.75 (m, 5H), 4.58 (d, *J* = 11.3 Hz, 1H), 4.32–4.27 (m, 1H), 3.99–3.90 (m, 2H), 3.85–3.71 (m, 3H), 3.67–3.36 (m, 14H), 3.31–3.20 (m, 3H), 2.40 (ddd, *J*_1_ = *J*_2_ = 4.5 Hz, *J*_3_ = 13.1, Hz, 1H), 2.30–2.23 (m, 1H), 2.12–2.02 (m, 3H), 1.64 (ddd, *J*_1_ = *J*_2_ = *J*_3_ = 13.0 Hz, 1H). ^13^C NMR (100 MHz, CDCl_3_) δ138.51, 137.84, 137.46, 137.24, 136.76, 128.83, 128.61, 128.58, 128.45, 128.36, 128.18, 128.15, 127.91, 127.89, 127.50, 127.29, 127.13, 126.24, 125.37, 101.26, 97.08, 96.28, 82.61, 81.40, 80.12, 79.59, 78.34, 77.97, 77.70, 77.23, 77.02, 76.81, 75.09, 74.93, 74.49, 73.3, 70.85, 69.59, 68.54, 66.65, 62.85, 61.46, 60.12, 59.3, 57.7, 53.62, 51.28, 32.17, 29.69. HRMS (ESI/APCI) calculated for (C_59_H_68_N_13_O_12_) [M + H^+^]: 1150.5105, found: 1150.5063.
*2′-O-(2-n-Pentylamino-ethyl)-1,3,6′,3″-tetraazido-4″,6″-O-benzylidene-5,3′,4′,2″-tetra-O-benzylkanamycin A* (**22d**): This compound was synthesized through reductive amination by coupling **17** with n-pentylamine with the same procedure as described in the preparation of **22a**. 64% yield, colorless semisolid. ^1^H NMR (400 MHz, CDCl_3_) δ 7.45–7.21 (m, 20H), 7.18–7.14 (m, 4H), 7.06 (t, *J* = 7.3 Hz, 1H), 5.56–5.54 (m, 2H), 5.30 (s, 1H), 5.05 (d, *J* = 12.1 Hz, 1H), 4.95 (d, *J* = 12.1 Hz, 1H), 4.86–4.75 (m, 5H), 4.57 (d, *J* = 11.2 Hz, 1H), 4.29–4.23 (m, 1H), 4.01 (dd, *J*_1_ = 4.8, *J_2_* = 10.1 Hz, 1H), 3.92 (t, *J* = 9.3 Hz, 1H), 3.89–3.81 (m, 2H), 3.77 (t, *J* = 9.3 Hz, 1H), 3.70–3.20 (m, 13H), 2.54–2.51 (m, 1H), 2.46–2.22 (m, 4H), 1.65 (ddd, *J*_1_ = *J*_2_ = *J*_3_ = 12.6 Hz, 1H), 1.33–1.06 (m, 6H), 0.84 (t, *J* = 7.2 Hz, 3H). ^13^C NMR (100 MHz, CDCl_3_) δ 138.34, 137.83, 137.35, 137.17, 136.71, 128.84, 128.55, 128.43, 128.38, 128.14, 127.92, 127.85, 127.60, 127.43, 127.32, 126.24, 125.49, 101.33, 96.80, 96.28, 82.58, 81.18, 80.19, 79.54, 78.30, 78.00, 77.83, 77.53, 75.33, 74.90, 74.64, 73.27, 71.25, 70.83, 68.54, 62.87, 61.39, 60.11, 59.46, 51.28, 49.61, 49.14, 32.21, 29.37, 29.16, 22.49, 14.04. HRMS (ESI/APCI) calculated for (C_60_H_72_N_13_O_11_) [M + H^+^]: 1150.5469, found: 1150.5430.
*2′-O-(2-Cyclohexylamino-ethyl)-1,3,6′,3″-tetraazido-4″,6″-O-benzylidene-5,3′,4′,2″-tetra-O-benzylkanamycin A* (**22e**): This compound was synthesized through reductive amination by coupling **17** with cyclohexylamine with the same procedure as described in the preparation of **22a**. 52% yield, colorless semisolid. ^1^H NMR (400 MHz, CDCl_3_) δ 7.45–7.22 (m, 20H), 7.19–7.15 (m, 4H), 7.08 (t, *J* = 7.3 Hz, 1H), 5.55–5.53 (m, 2H), 5.32 (s, 1H), 5.02 (d, *J* = 12.1 Hz, 1H), 4.94 (d, *J* = 12.0 Hz, 1H), 4.86–4.73 (m, 5H), 4.56 (d, *J* = 11.3 Hz, 1H), 4.26–4.20 (m, 1H), 4.01 (dd, *J*_1_ = 4.8 Hz, *J*_2_ = 10.1Hz, 1H), 3.94–3.83 (m, 3H), 3.78 (t, *J* = 9.3 Hz, 1H), 3.69–3.31 (m, 12H), 3.25 (t, *J* = 9.8 Hz, 1H), 2.64–2.50 (m, 2H), 2.38 (ddd, *J*_1_ = *J*_2_ = 4.4 Hz, *J*_3_ = 13.0 Hz, 1H), 2.19–2.12 (m, 1H), 1.71–1.51 (m, 4H), 1.12–0.80 (m, 6H). ^13^C NMR (100 MHz, CDCl_3_) δ 138.36, 137.83, 137.44, 137.26, 136.80, 128.95, 128.66, 128.52, 128.24, 128.02, 127.97, 127.74, 127.49, 126.32, 125.74, 101.44, 96.74, 96.36, 82.55, 81.03, 80.11, 79.63, 78.43, 78.12, 77.94, 77.83, 75.26, 74.89, 74.68, 73.37, 71.05, 68.63, 62.97, 61.47, 60.16, 59.60, 56.70, 51.37, 45.57, 32.26, 31.88, 25.64, 24.86. HRMS (ESI/APCI) calculated for (C_61_H_72_N_13_O_11_) [M + H^+^]: 1162.5469, found: 1162.5486.

General procedure for the deprotection of compounds **18**, **19**, and **22a**–**22e**: The starting compound (0.03–0.08 mmol) was dissolved in the mixture of tetrahydrofuran (2 mL) and water (1 mL). Then, 50 mg of sodium hydroxide was added. The mixture was stirred for 10 min at room temperature, then 1 mL of trimethylphosphine solution in tetrahydrofuran (1 M) was added. After TLC showed the reaction was completed, the mixture was concentrated and the residue was passed through a short column (silica gel) with eluents as the following: methanol (30 mL), and methanol/ammonia solution in methanol (7 M) (50 mL/5 mL). The proper fractions were collected, combined, and concentrated. The resulting amine was then dissolved in the mixture of methanol (5 mL) and tetrahydrofuran (1 mL), and the pH value of the resulting solution was adjusted to 3–4 with hydrochloric acid (1 M). Then, Pd/C (10%, 50 mg) was added. The mixture was subjected to hydrogenolysis for 2–5 days. After TLC showed that only one spot appeared, the mixture was filtered through a pad of celite. To the filtrate was added 0.1 mL of triethylamine, and then a small volume of silica gel was added. The solvent was removed, and the resulting mixture was transferred to a silica gel column. After eluting the column with methanol (50 mL), methanol/concentrated aqueous ammonia (100 mL/10 mL), and methanol/concentrated aqueous ammonia (50 mL/10 mL), the fractions with the desired product were collected and combined. After removal of solvent, the gross product was redissolved in acetic acid solution in water (0.05 mol/L, 5 mL) and the resulting solution was freeze-dried. Thus, we obtained the final products **20**, **21**, and **23a**–**23e**.

*2′-O-(2-Hydroxyethyl)-kanamycin A* (**20**): 78% yield, white amorphous powder. ^1^H NMR (600 MHz, D_2_O) δ 5.70 (d, *J* = 3.8 Hz, 1H), 5.10 (d, *J* = 3.6 Hz, 1H), 3.99–3.95 (m, 1H), 3.93–3.80 (m, 8H), 3.77–3.70 (m, 4H), 3.67 (t, *J* = 10.1 Hz, 1H), 3.58–3.45 (m, 4H), 3.43–3.36 (m, 2H), 3.15 (dd, *J*_1_ = 8.1 Hz, *J_2_* = 13.4 Hz, 1H), 2.50 (ddd, *J*_1_ = *J_2_* = 4.2 Hz, *J*_3_ = 12.5 Hz, 1H), 1.94 (s, 12H), 1.90 (ddd, *J*_1_ = *J*_2_ = *J*_3_ = 12.5 Hz, 1H). ^13^C NMR (150 MHz, D_2_O) δ 180.83, 101.36, 95.18, 84.83, 79.81, 78.73, 73.94, 73.51, 73.34, 72.56, 71.64, 69.27, 68.88, 66.16, 61.30, 60.67, 55.65, 50.51, 48.36, 41.04, 28.46, 23.19. HRMS (ESI/APCI) calculated for (C_20_H_41_N_4_O_12_) [M + H]^+^ requires *m/z* 529.2715, found *m/z* 529.2734.
*2′-O-(2-Aminoethyl)-kanamycin A* (**21**): 58% yield, white amorphous powder. ^1^H NMR (600 MHz, D_2_O) δ 5.83 (d, *J* = 3.8 Hz, 1H), 5.11 (d, *J* = 3.7 Hz, 1H), 4.09–4.06 (m, 1H), 3.95–3.87 (m, 7H), 3.83 (dd, *J*_1_ = 2.2 Hz, *J*_2_ = 12.3 Hz, 1H), 3.77–3.73 (m, 2H), 3.67 (t, *J* = 10.1 Hz, 1H), 3.57–3.40 (m, 6H), 3.30–3.26 (m, 1H), 3.21–3.18 (m, 2H), 2.49 (ddd, *J*_1_ = *J_2_* = 4.1 Hz, *J*_3_ = 12.6 Hz, 1H), 1.93 (s, 15H), 1.89 (ddd, *J*_1_ = *J_2_* = *J*_3_ = 12.7 Hz, 1H). ^13^C NMR (150 MHz, D_2_O) δ 181.37, 101.31, 95.57, 84.95, 79.72, 78.29, 74.25, 73.65, 71.76, 71.69, 69.30, 68.84, 67.98, 66.27, 60.78, 55.67, 50.44, 48.92, 40.98, 39.94, 28.89, 23.53. HRMS (ESI/APCI) calculated for (C_20_H_42_N_5_O_11_) [M + H]^+^ requires *m/z* 528.2875, found *m/z* 528.2892.
*2′-O-[2-(4-Mopholinyl)-ethyl]-kanamycin A* (**23c**): 62% yield, white amorphous powder. ^1^H NMR (600 MHz, D_2_O) δ 5.89 (d, *J* = 3.7 Hz, 1H), 5.11 (d, *J* = 3.6 Hz, 1H), 4.17 (ddd, *J*_1_ = *J*_2_ = 4.4 Hz, *J*_3_ = 12.2 Hz, 1H), 4.02–3.73 (m, 14H), 3.67 (t, *J* = 10.1 Hz, 1H), 3.58–3.39 (m, 12H), 3.19 (dd, *J*_1_ = 7.7 Hz, *J*_2_ = 13.4 Hz, 1H), 2.50 (ddd, *J*_1_ = *J*_2_ = 4.2 Hz, *J*_3_ = 12.5 Hz, 1H), 1.97–1.91 (m, 16H). ^13^C NMR (150 MHz, D_2_O) δ 180.26, 100.56, 94.57, 84.09, 78.86, 76.79, 73.67, 72.88, 71.06, 70.71, 68.53, 68.06, 65.43, 63.94, 63.70, 59.93, 56.19, 54.88, 51.76, 49.59, 48.24, 40.22, 27.90, 22.56. HRMS (ESI/APCI) calculated for (C_24_H_48_N_5_O_12_) [M + H]^+^ requires *m/z* 598.3294, found *m/z* 598.3315.
*2′-O-[2-[(2-Aminoethyl)amino]-ethyl]-kanamycin A* (**23a**): 55% yield, white amorphous powder. ^1^H NMR (600 MHz, D_2_O) δ 5.84 (d, *J* = 3.7 Hz, 1H), 5.13 (d, *J* = 3.6 Hz, 1H), 4.09–4.05(m, 1H), 3.99–3.92 (m, 3H), 3.91–3.66 (m, 8H), 3.55–3.15 (m, 13H), 2.40 (ddd, *J*_1_ = *J*_2_ = 4.2 Hz, *J*_3_ = 12.7 Hz, 1H), 1.92 (s, 17H), 1.80 (ddd, *J*_1_ = *J*_2_ = *J*_3_ = 12.6 Hz, 1H). ^13^C NMR (150 MHz, D_2_O) δ 182.09, 101.16, 95.41, 85.46, 79.81, 79.43, 74.52, 73.57, 71.86, 71.78, 69.16, 68.97, 67.93, 66.37, 60.74, 55.70, 50.68, 49.06, 48.30, 45.48, 41.09, 37.26, 30.25, 23.95. HRMS (ESI/APCI) calculated for (C_22_H_47_N_6_O_11_) [M + H]^+^ requires *m/z* 571.3297, found *m/z* 571.3318
*2′-O-[2-[(2-Piperizinylethyl)amino]-ethyl]-kanamycin A* (**23b**): 60% yield, white amorphous powder. ^1^H NMR (600 MHz, D_2_O) δ 5.78 (d, *J* = 3.7 Hz, 1H), 5.10 (d, *J* = 3.6 Hz, 1H), 3.97-3.91 (m, 4H), 3.86-3.81 (m, 4H), 3.78–3.74 (m, 2H), 3.70–3.64 (m, 2H), 3.50–3.33 (m, 6H), 3.27 (t, *J* = 4.9 Hz, 4H), 3.17 (dd, *J*_1_ = 8.0 Hz, *J*_2_ = 13.4 Hz, 1H), 2.87–2.80 (m, 4H), 2.76–2.72 (m, 2H), 2.39 (ddd, *J*_1_ = *J*_2_ = 4.1 Hz, *J*_3_ = 12.7 Hz, 1H), 1.90 (s, 15H), 1.77 (ddd, *J*_1_ = *J*_2_ = *J*_3_ = 12.5 Hz, 1H). ^13^C NMR (150 MHz, D_2_O) δ 181.98, 101.17, 95.39, 85.46, 79.95, 79.54, 74.40, 73.54, 72.00, 71.76, 69.10, 68.93, 67.92, 66.28, 60.67, 57.12, 55.71, 50.68, 49.89, 48.94, 43.57, 41.06, 30.12, 23.87. HRMS (ESI/APCI) calculated for (C_24_H_49_N_6_O_11_) [M + H]^+^ requires *m/z* 597.3454, found *m/z* 597.3473.
*2′-O-[2-(n-Pentylamino)-ethyl]-kanamycin A* (**23d**): 68% yield, white amorphous powder. ^1^H NMR (600 MHz, D_2_O) δ 5.88 (d, *J* = 3.5 Hz, 1H), 5.14 (d, *J* = 3.4 Hz, 1H), 4.14–4.11 (m, 1H), 4.00–3.84 (m, 8H), 3.78–3.75 (m, 2H), 3.69 (t, *J* = 10.1 Hz, 1H), 3.57–3.41 (m, 6H), 3.36–3.33 (m, 1H), 3.29–3.25 (m, 1H), 3.22–3.18 (m, 1H), 3.09 (t, J = 7.7 Hz, 2H), 2.46 (ddd, *J*_1_ = *J*_2_ = 4.5 Hz, *J*_3_ = 12.5 Hz, 1H), 1.93 (s, 17H), 1.90 (ddd, *J*_1_ = *J*_2_ = *J*_3_ = 12.5 Hz, 1H), 1.73–1.68 (m, 2H), 1.39–1.32 (m, 4H), 0.90 (t, *J* = 6.9 Hz, 3H). ^13^C NMR (150 MHz, D_2_O) δ 181.71, 101.27, 95.52, 85.16, 79.68, 78.52, 74.64, 73.65, 71.87, 71.62, 69.24, 68.90, 66.66, 66.30, 60.77, 55.68, 50.57, 49.12, 48.34, 47.56, 41.09, 29.51, 28.57, 25.87, 23.71, 22.21, 13.77. HRMS (ESI/APCI) calculated for (C_25_H_52_N_5_O_11_) [M + H]^+^ requires *m/z* 598.3658, found *m/z* 598.3678.
*2′-O-[2-(Cyclohexylamino)-ethyl]-kanamycin A* (**23e**): 64% yield, white amorphous powder. ^1^H NMR (600 MHz, D_2_O) δ 5.84 (d, *J* = 3.7 Hz, 1H), 5.11 (d, *J* = 3.7 Hz, 1H), 4.12–4.09 (m, 1H), 3.96–3.80 (m, 8H), 3.77–3.65 (m, 3H), 3.55–3.34 (m, 7H), 3.29–3.25 (m, 1H), 3.21–3.12 (m, 2H), 2.42 (ddd, *J*_1_ = *J*_2_ = 4.5 Hz, *J*_3_ = 12.4 Hz, 1H), 2.08 (br, 2H), 1.92 (s, 12H), 1.85–1.79 (m, 3H), 1.69–1.64 (m, 1H), 1.40–1.27 (m, 4H), 1.21–1.14 (m, 1H). ^13^C NMR (150 MHz, D_2_O) δ 181.90, 101.25, 95.59, 85.29, 79.65, 79.08, 74.59, 73.63, 71.79, 71.63, 69.21, 68.89, 66.87, 66.28, 60.74, 58.02, 55.69, 50.59, 49.09, 44.59, 41.01, 29.81, 29.70, 29.50, 25.19, 24.68, 24.65, 23.88. HRMS (ESI/APCI) calculated for (C_26_H_52_N_5_O_11_) [M + H]^+^ requires *m/z* 610.3658, found *m/z* 610.3680.

### 3.2. Bioassay

Ten bacterial strains were selected to evaluate the minimal inhibitory concentration (MIC) of compounds. All newly synthesized compounds were tested in the form of acetate. Corning 96-well plates were utilized for this test. Briefly, tested strains were seeded into 200 μL Mueller–Hinton (MH) broth per well with a concentration of 10^5^ CFU/mL. Subsequently, an aliquot of sample stock was added, with a series of final concentrations of 1–128 μg/mL. All of the mixtures were incubated at 37 °C for 24 h. The MICs were determined by measuring the optical density at 600 nm. The sterilized water (0 μg/mL) was used as the control; all of the tests were performed in triplicate.

## 4. Conclusions

According to the fact that some modified products of aminoglycosides by AAC(2′) remain active, the possible reason was analyzed by means of some structural biology data. It was deduced that the 2′-position of neamine-containing aminoglycosides is a proper position for modification. Based on this hypothesis, we designed, synthesized, and evaluated a series of 2′-modified derivatives of kanamycin A. As expected, all derivatives exhibited moderate to good antibacterial activity. The structure–activity relationship showed that the feature of the introduced groups on the 2′-position, including the number of amino groups, rigidity, hydrophobicity, and bulk, had a mild impact on activity. All of these results were believed to be attributed to the fact that 2′-substituents point to a vacant space. On the other hand, proactive chemical modification on the 2′-position may bring some benefits to fight against drug-resistant bacteria, but cannot achieve a strong and broad-spectrum effect.

## Figures and Tables

**Table 1:** The results of antibacterial activities of synthesized compounds.

	MIC (µg/mL)									
Compd	*E. coli*		*S. aureus*			*K. pneumoniae*		*E. faecalis*	*P. aeruginosa*	
	ATCC 35218	ATCC 25922	ATCC 29213	ATCC 25923	ATCC 33591	ATCC 700603	ATCC 13883	ATCC 29212	ATCC 27853	PAO1
**Kan**	2	4	2	4	64	16	1	64	>128	8
**20**	4	32	1	4	64	8	2	64	>128	16
**21**	2	16	0.5	0.5	8	2	1	64	128	2
**23a**	4	>128	0.5	4	8	4	2	64	128	16
**23b**	4	64	1	4	128	8	4	128	>128	>128
**23c**	8	32	0.5	4	128	8	4	64	>128	128
**23d**	8	64	1	8	32	16	4	64	>128	128
**23e**	2	16	1	4	8	4	1	64	>128	16

## Data Availability

The data presented in this study are available on request from the corresponding author.

## Supplementary Materials

The following supporting information can be downloaded at: https://www.mdpi.com/article/10.3390/molecules27217482/s1, NMR Spectra.
