# Supplementary material for: Design, Synthesis, and Bioassay of 2′-Modified Kanamycin A
Source: Molecules. 2022 Nov 2;27(21):7482. doi: 10.3390/molecules27217482 (PMC9654810; doi:10.3390/molecules27217482)
Supplement: Supplementary file 1 [file molecules-27-07482-s001.zip › molecules-2000565-supplementary.pdf]

Design, synthesis, and bioassay of 2’-modified kanamycin A

YAN Ribai [1, *], LI Xiaonan [1], LIU Yuheng [2], YE Xinshan [1, *]

NMR Spetra

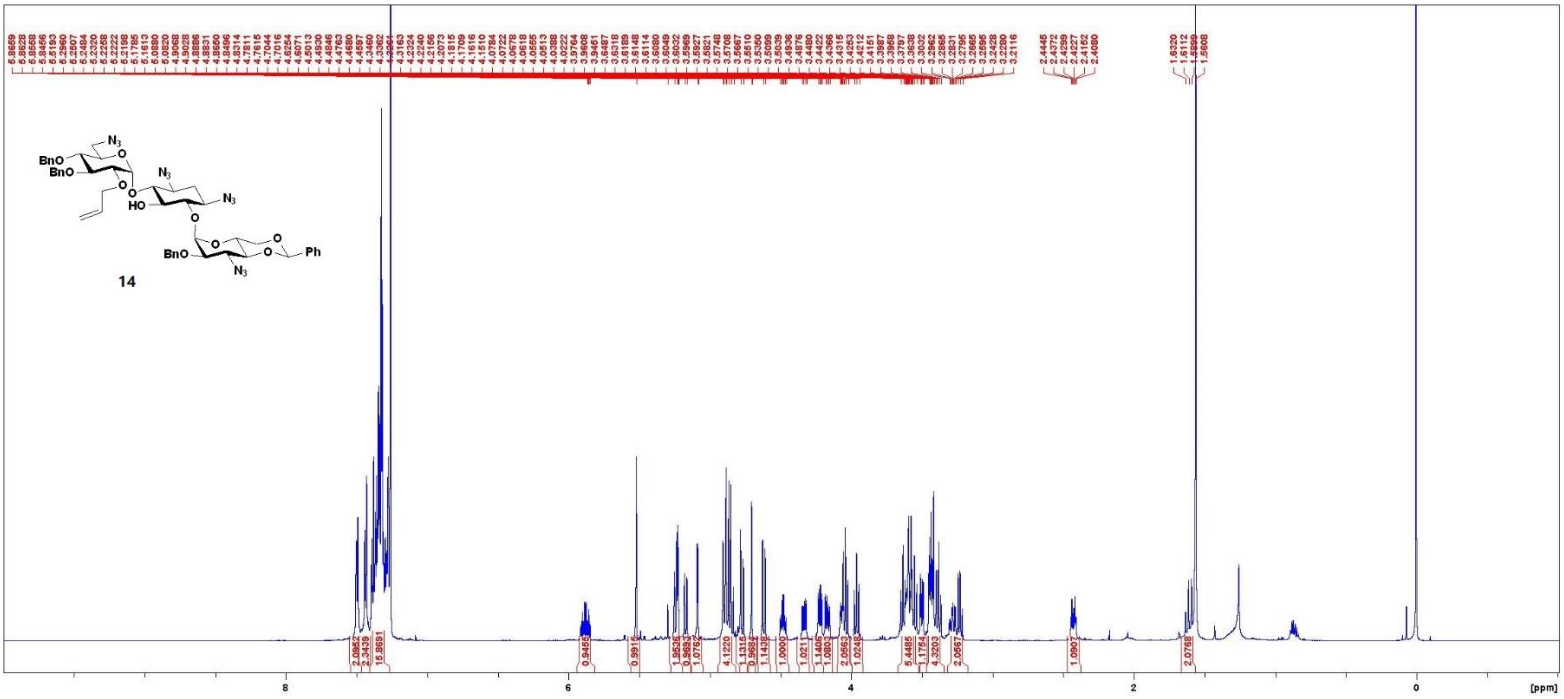
BnO
BnO
N3
N3
HO
N3
O
BnO
N3
Ph
14
8
6
4
2
0
[ppm]

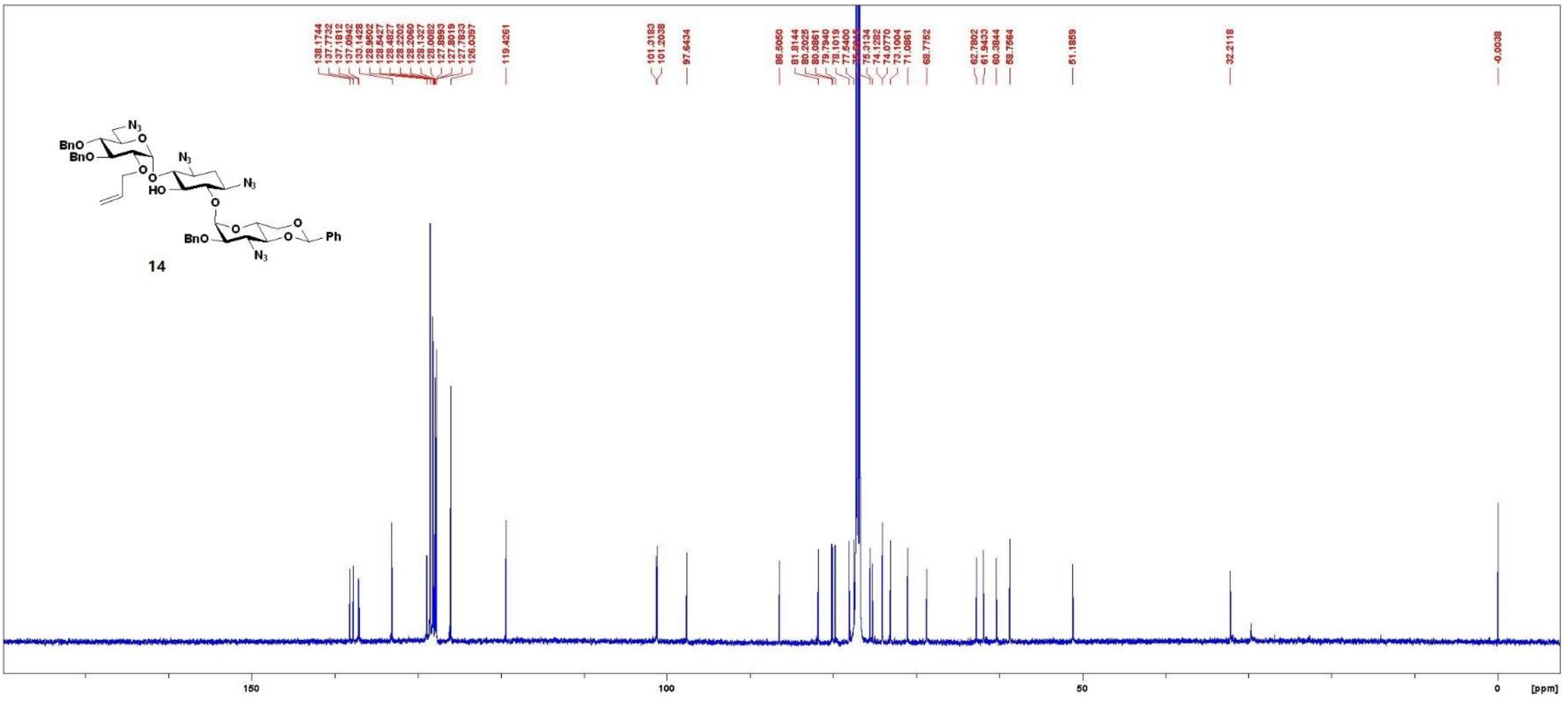
BnO
BnO
N3
N3
HO
N3
O
BnO
N3
Ph
14
150
100
50
0
[ppm]

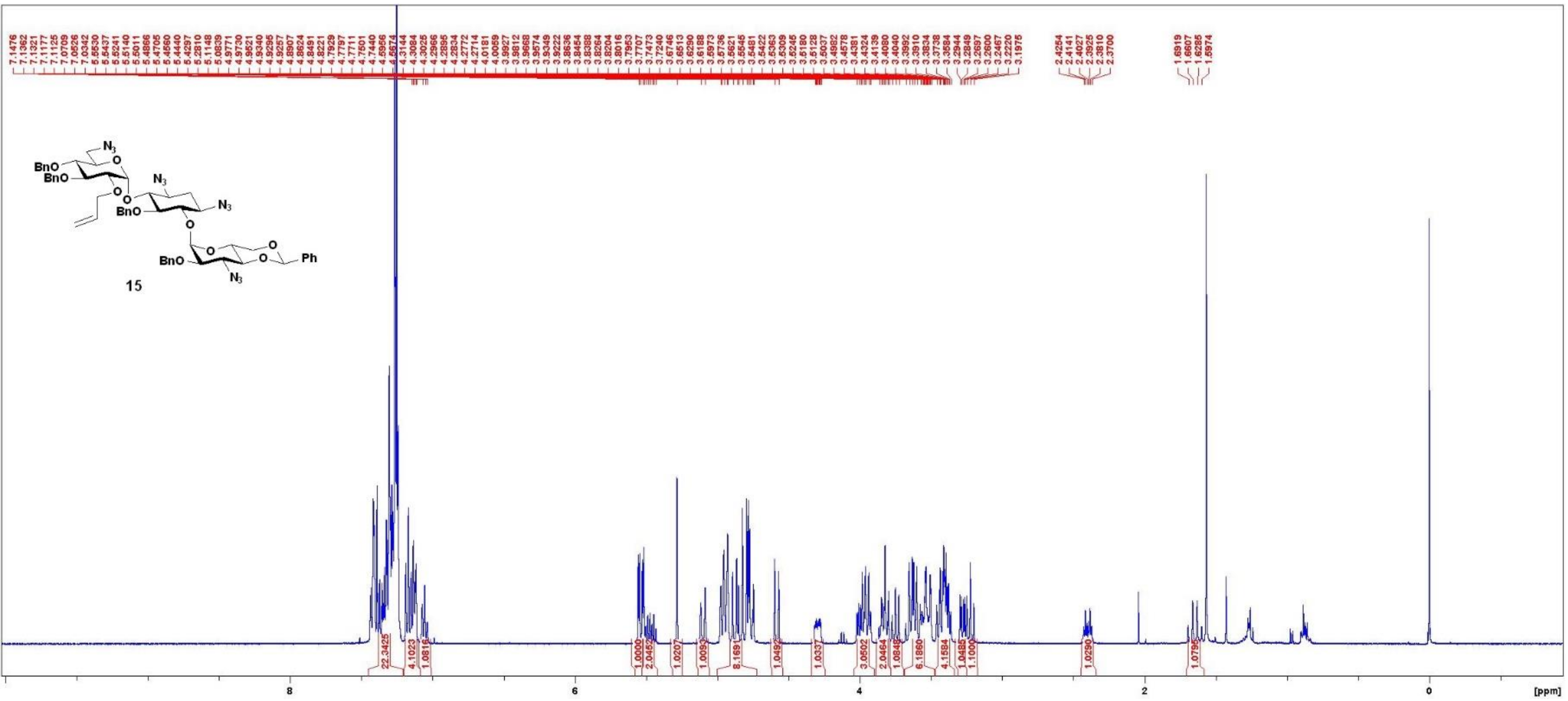
BnO
BnO
N3
BnO
N3
N3
BnO
N3
Ph
15
8
6
4
2
0
[ppm]

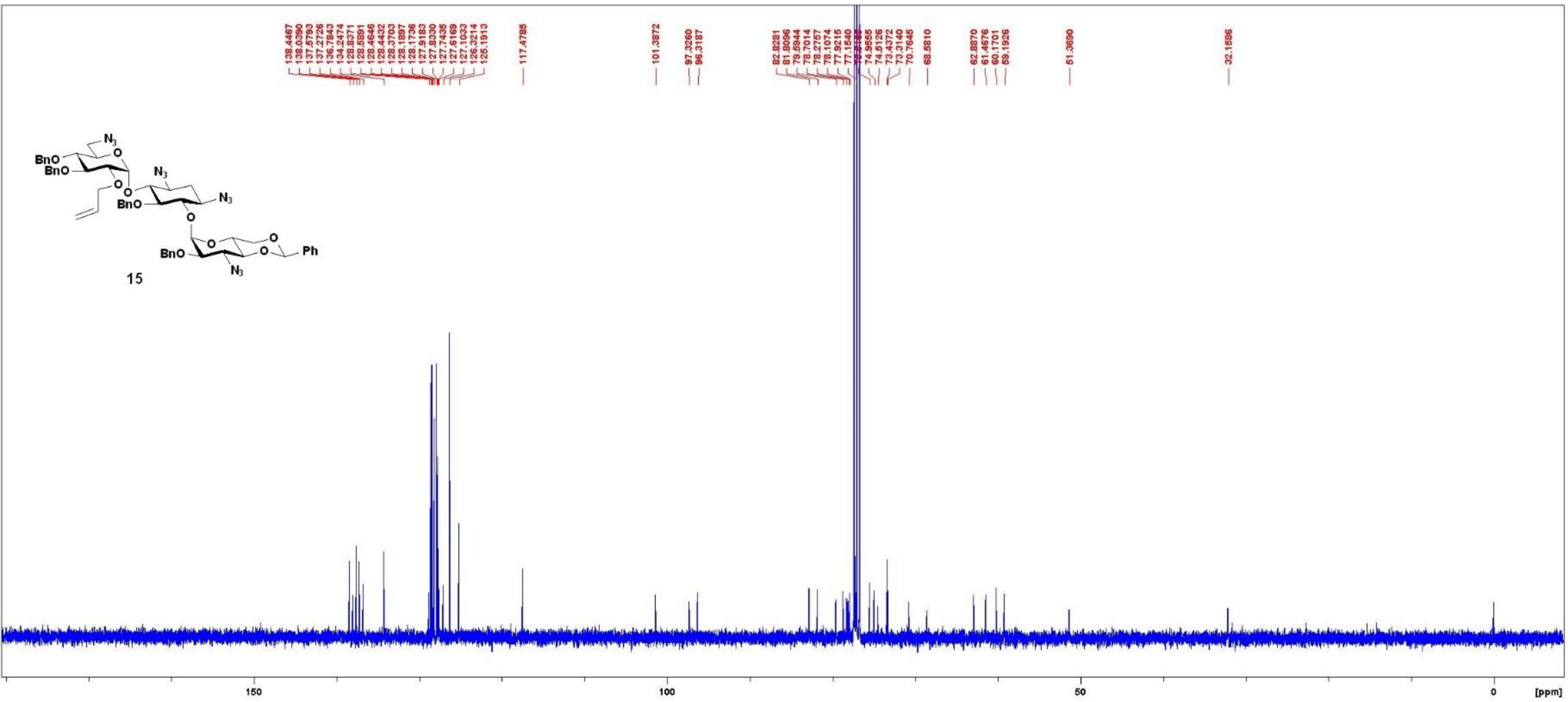
BnO
BnO
N3
BnO
N3
N3
BnO
N3
Ph
15
150
100
50
0
[ppm]

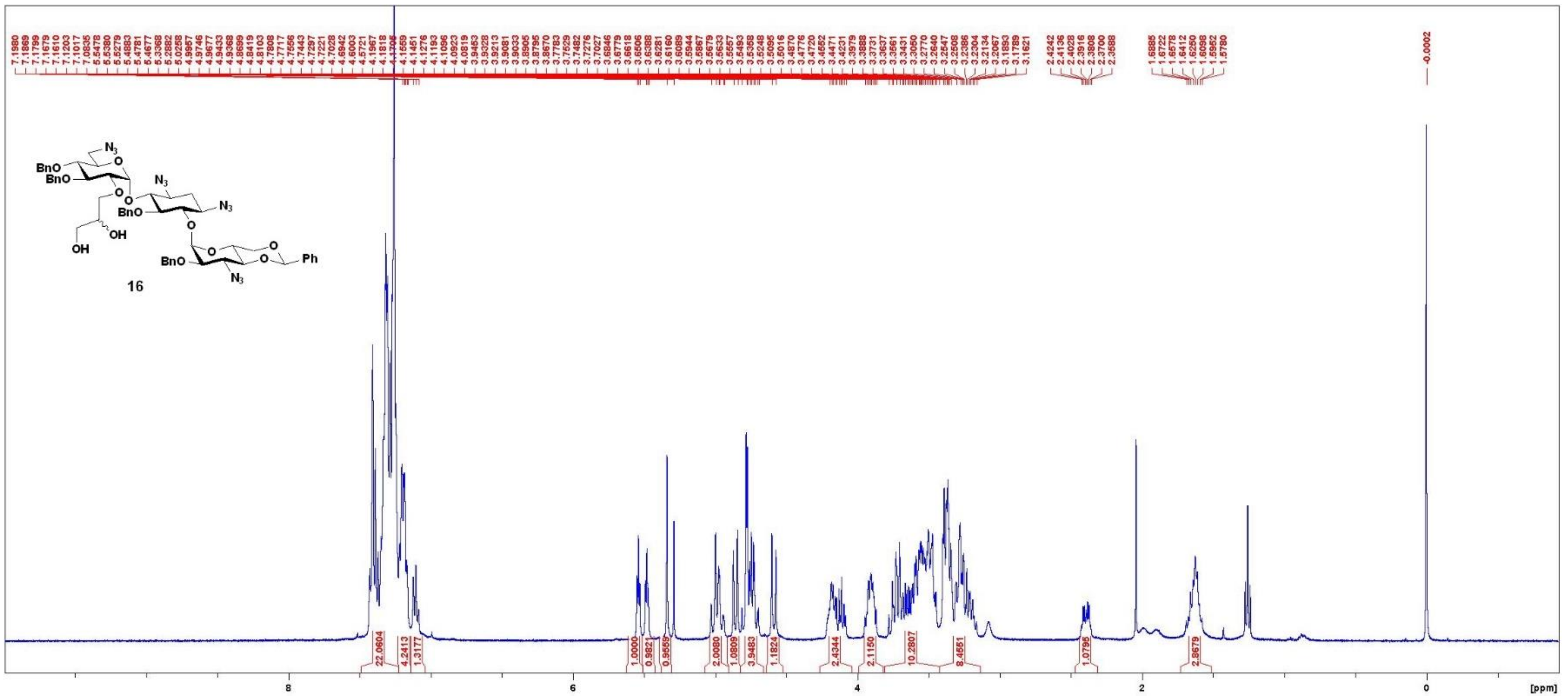

BnO
BnO
N3
OH
OH
BnO
N3
N3
BnO
N3
Ph
16
8
6
4
2
0
[ppm]

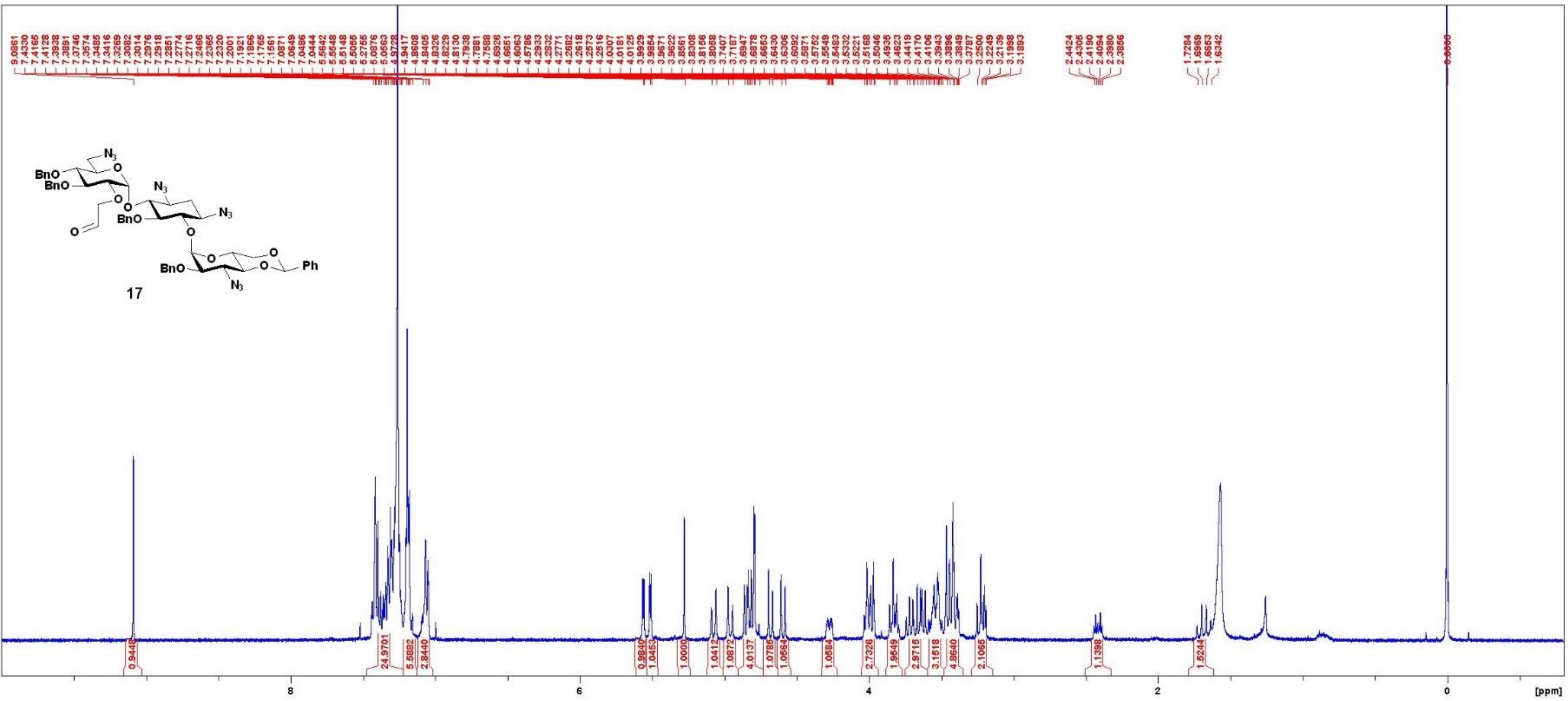

BnO
BnO
N3
O
BnO
N3
N3
BnO
N3
Ph
17
8
6
4
2
0
[ppm]

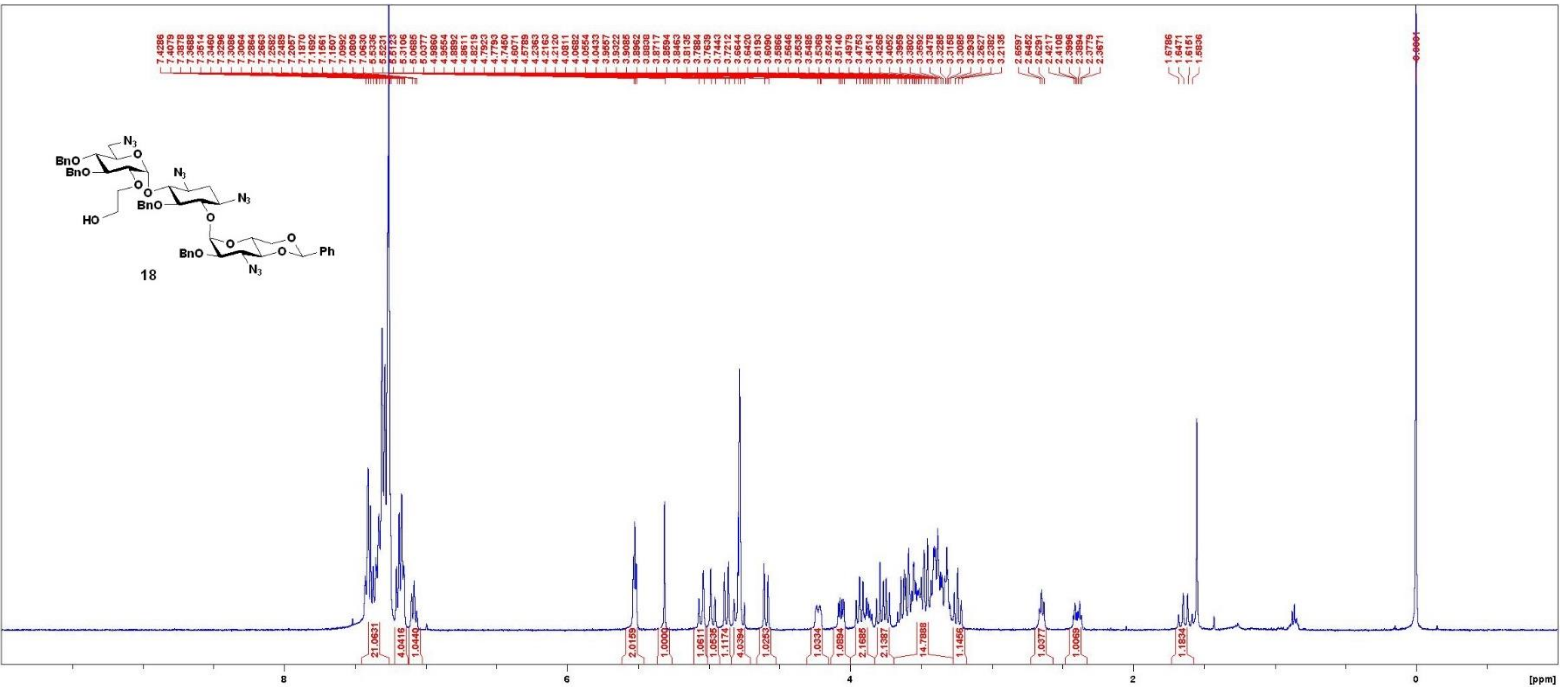
BnO
BnO
N3
N3
N3
BnO
HO
BnO
N3
Ph
18
8
6
4
2
0
[ppm]

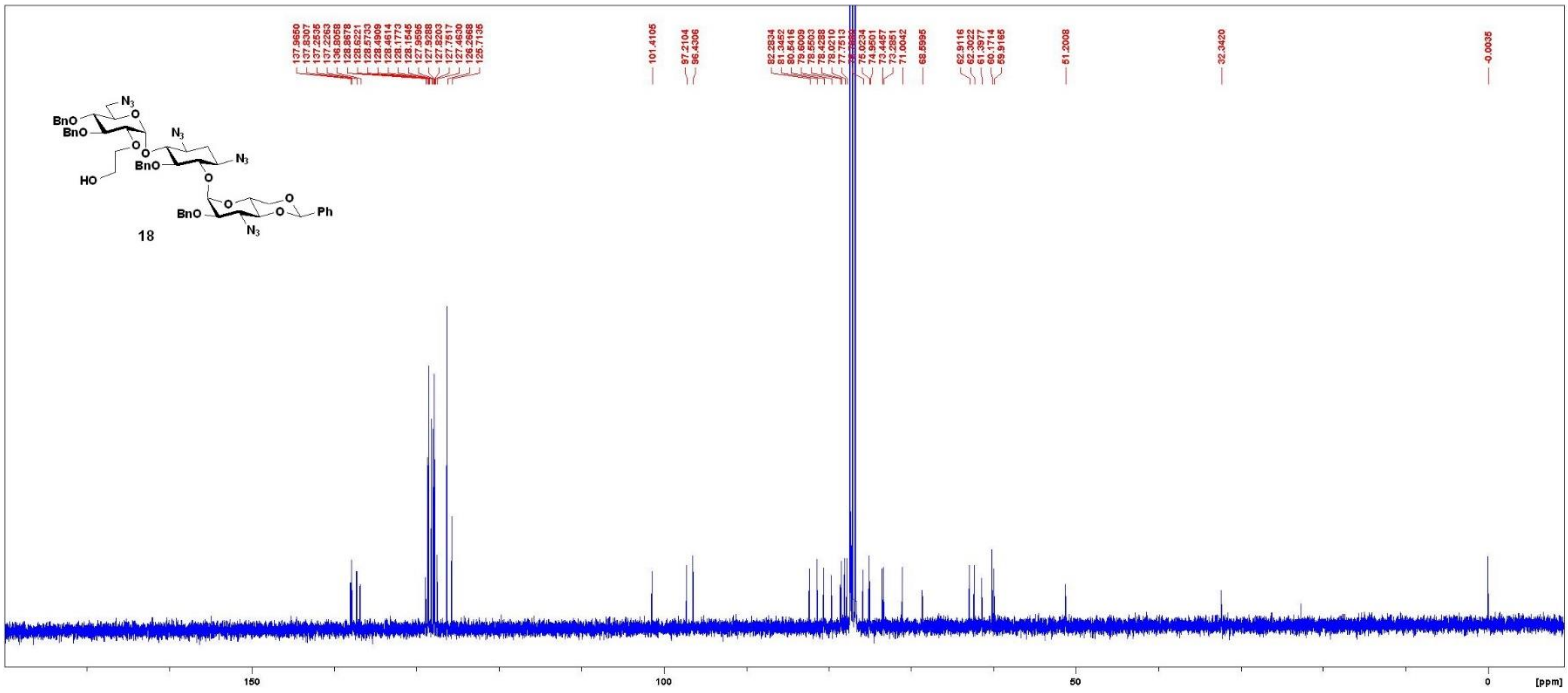
BnO
BnO
N3
N3
N3
BnO
HO
BnO
N3
Ph
18
101.4105
97.2104
96.4306
68.5995
51.2008
32.3420
-0.0035
150
100
50
0
[ppm]

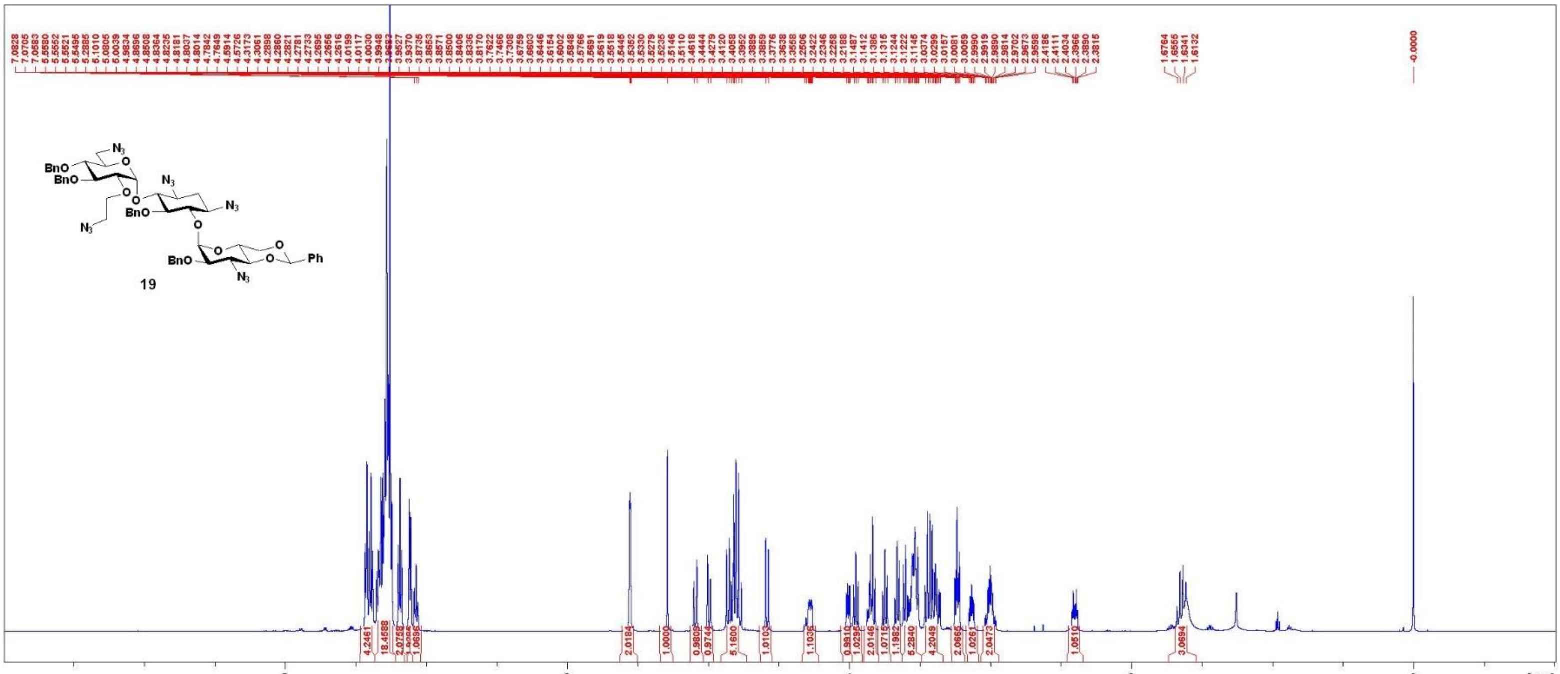

BnO
BnO
N3
N3
BnO
N3
BnO
N3
O
Ph
N3
19
8
6
4
2
0
[ppm]

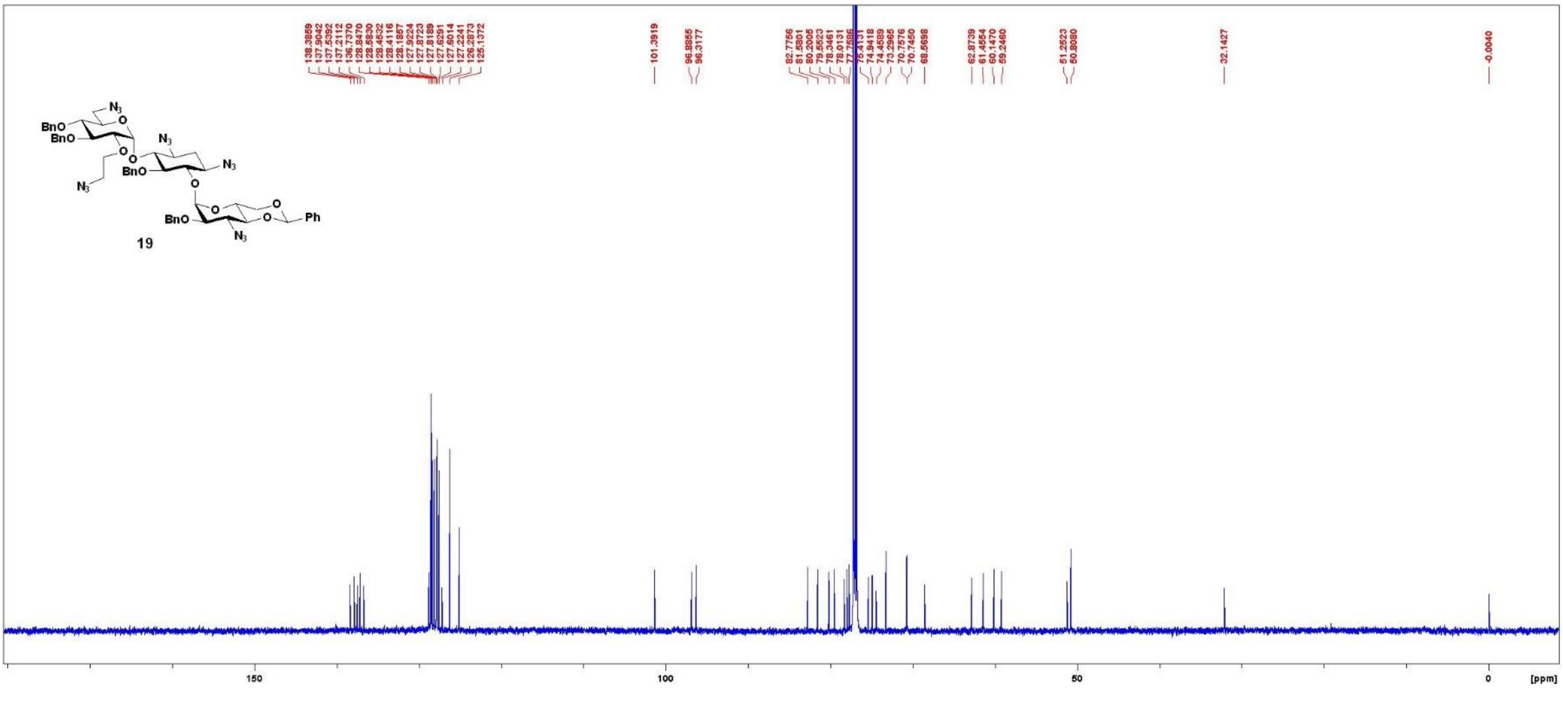

138.3859
137.9042
137.5392
137.2112
136.7370
128.8470
128.5830
128.4532
128.4116
128.1667
127.9224
127.8723
127.8189
127.6291
127.6014
127.2241
126.2873
126.1372
101.3919
96.8855
96.3177
82.7756
81.5801
80.2005
79.5523
78.3461
78.0131
77.7596
76.4131
74.9418
74.4589
73.2965
70.7576
70.7450
68.5698
62.8739
61.4554
60.1470
59.2460
51.2523
50.8080
32.1427
-0.0040
BnO
BnO
N3
N3
BnO
N3
BnO
N3
O
Ph
N3
19
150
100
50
0
[ppm]

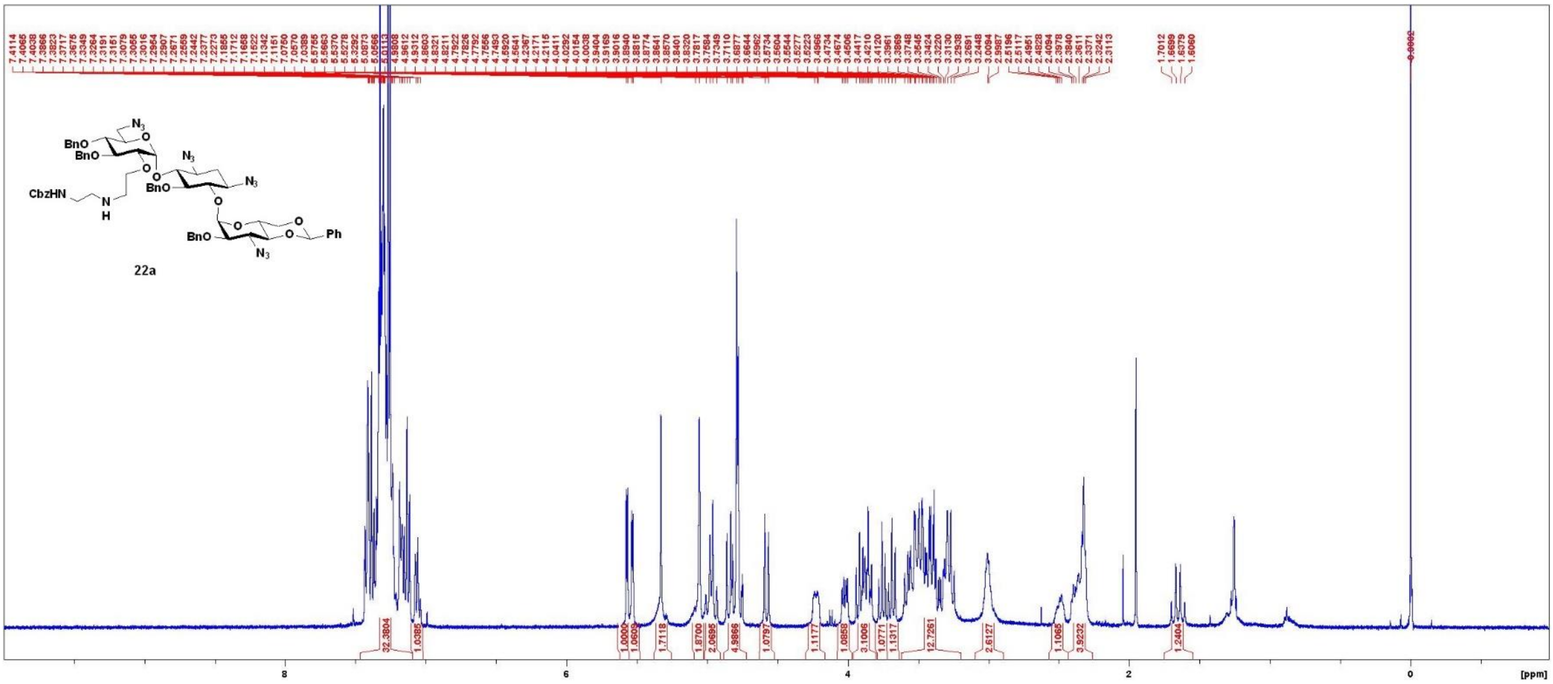
BnO
BnO
N3
BnO
N3
N3
CbzHN
N
H
BnO
N3
Ph
22a
8
6
4
2
0
[ppm]

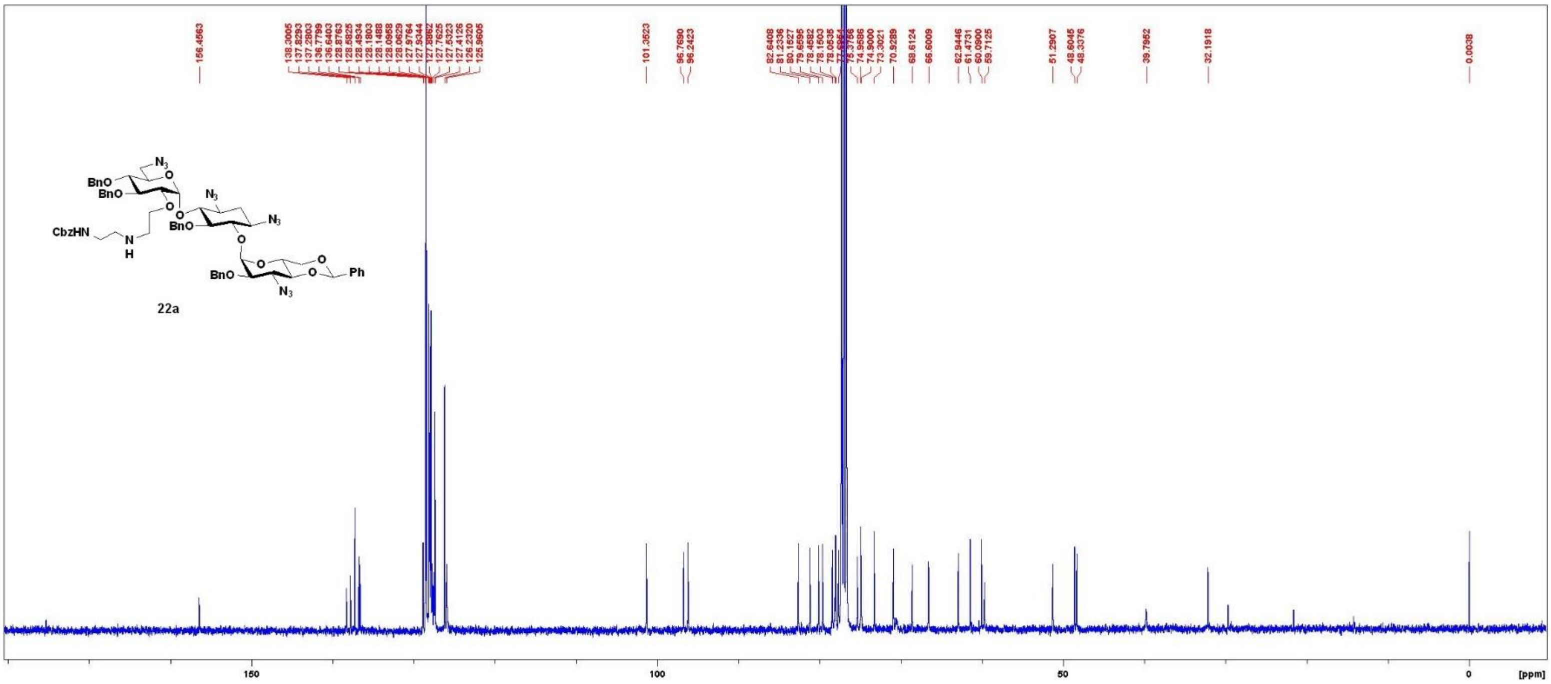
BnO
BnO
N3
BnO
N3
N3
CbzHN
N
H
BnO
N3
Ph
22a
150
100
50
0
[ppm]

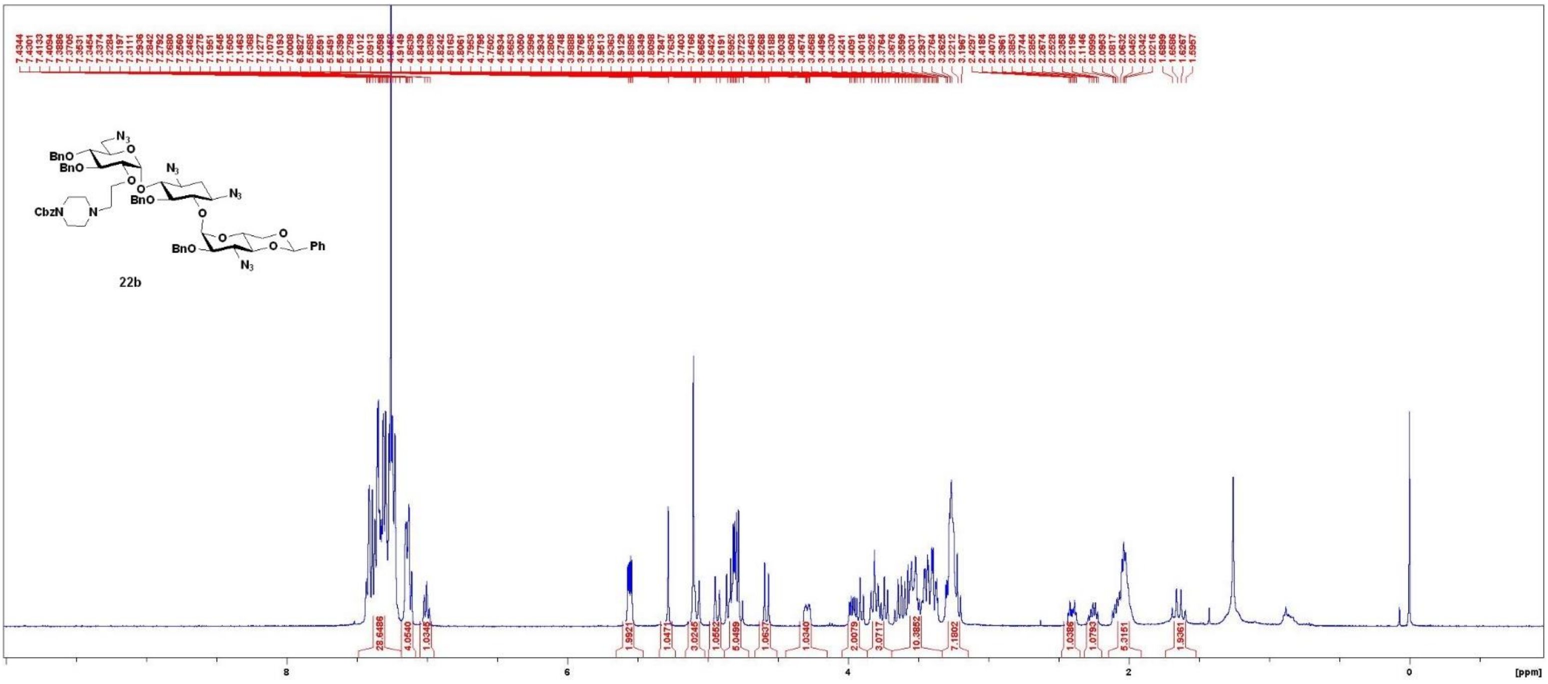
22b
8
6
4
2
0
[ppm]

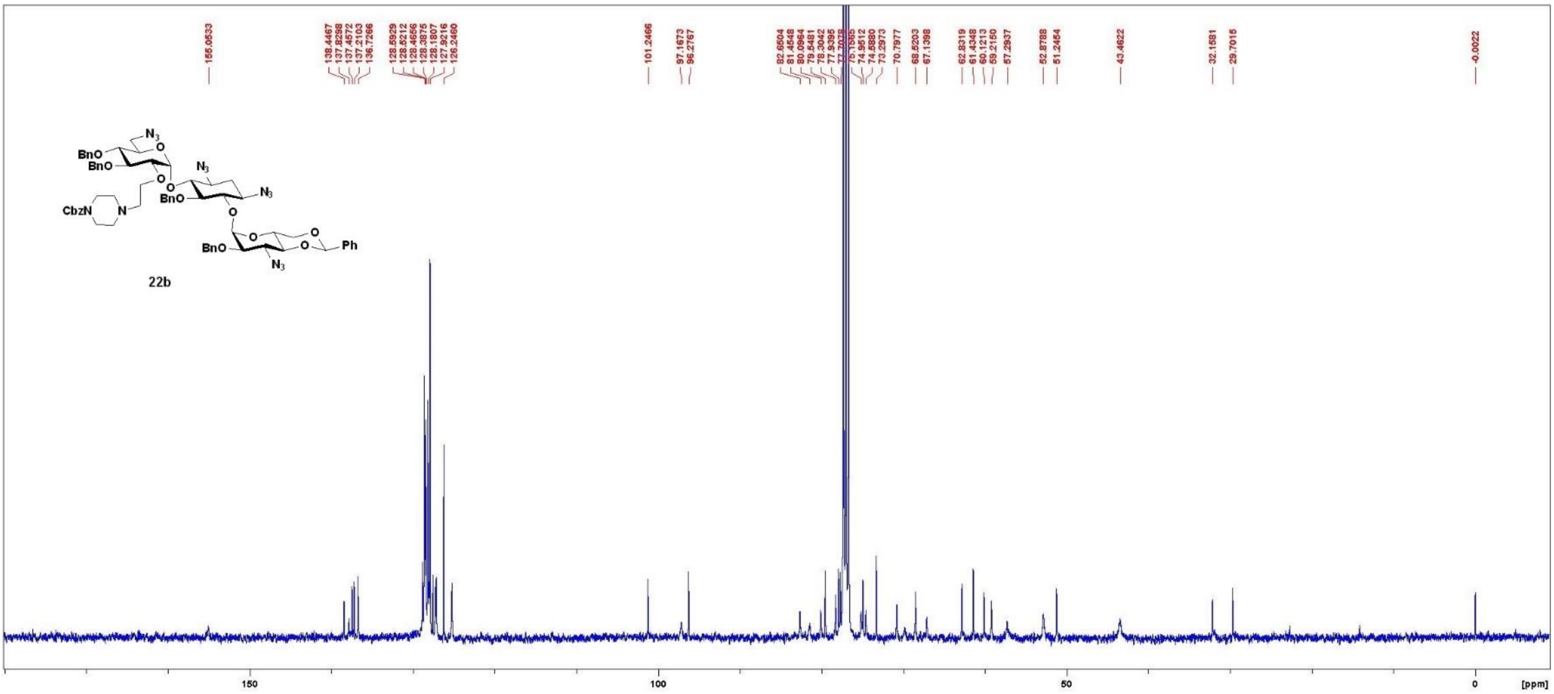
22b
150
100
50
0
[ppm]

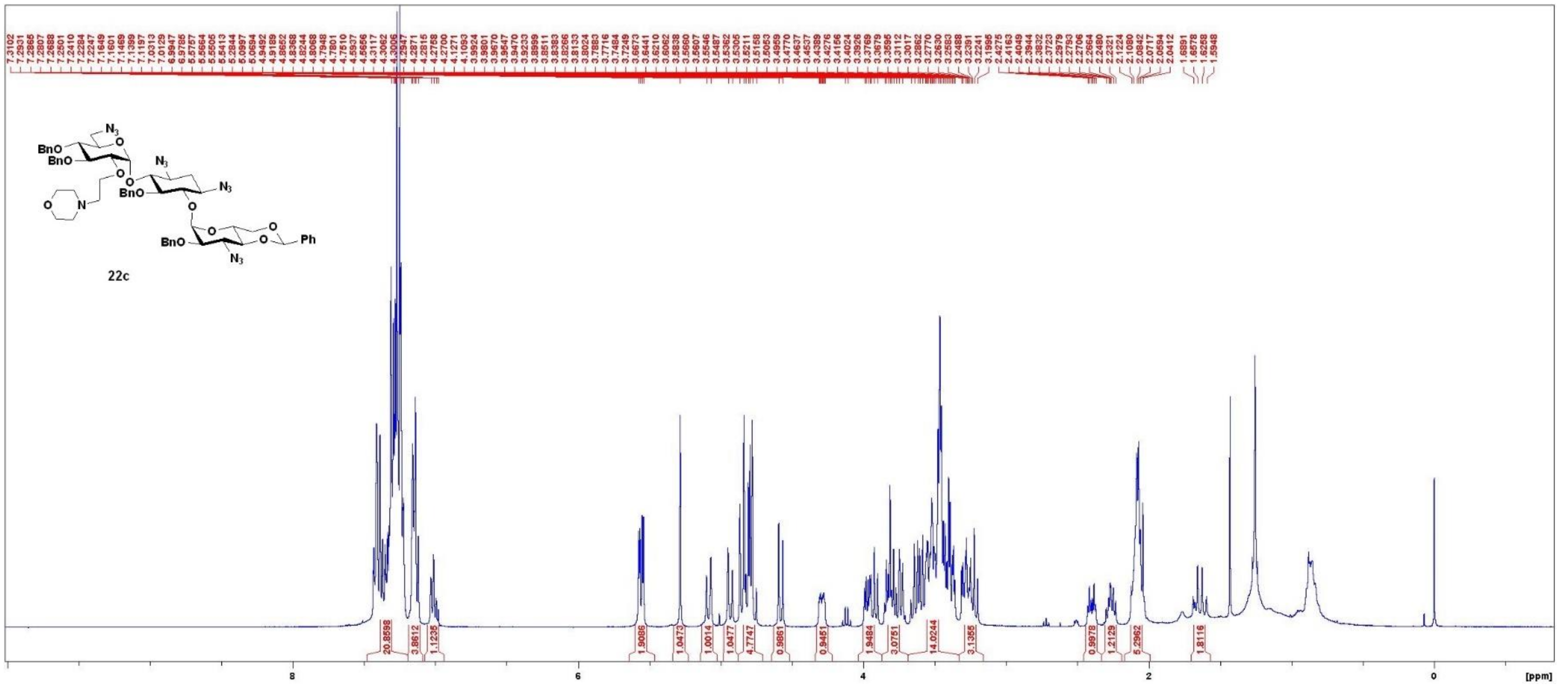
22c
[ppm]

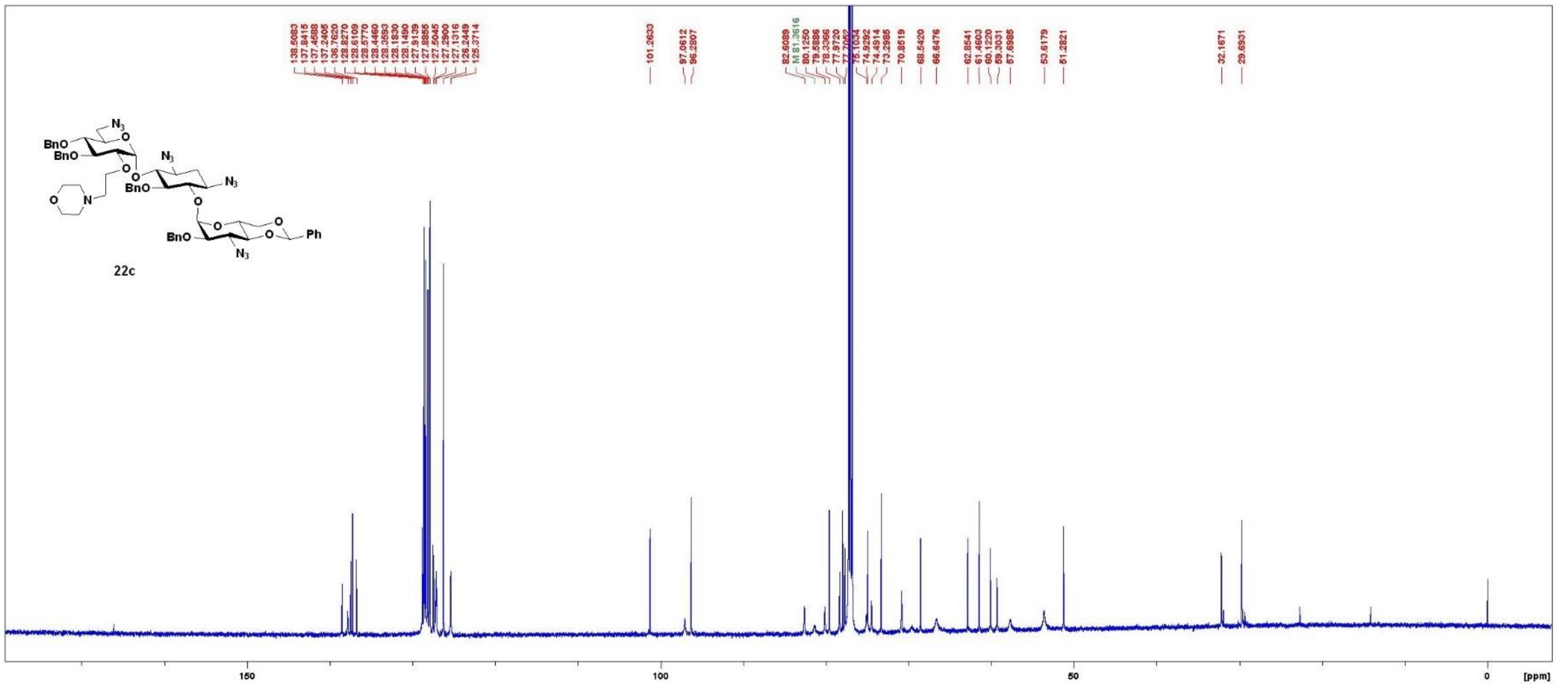
22c
[ppm]

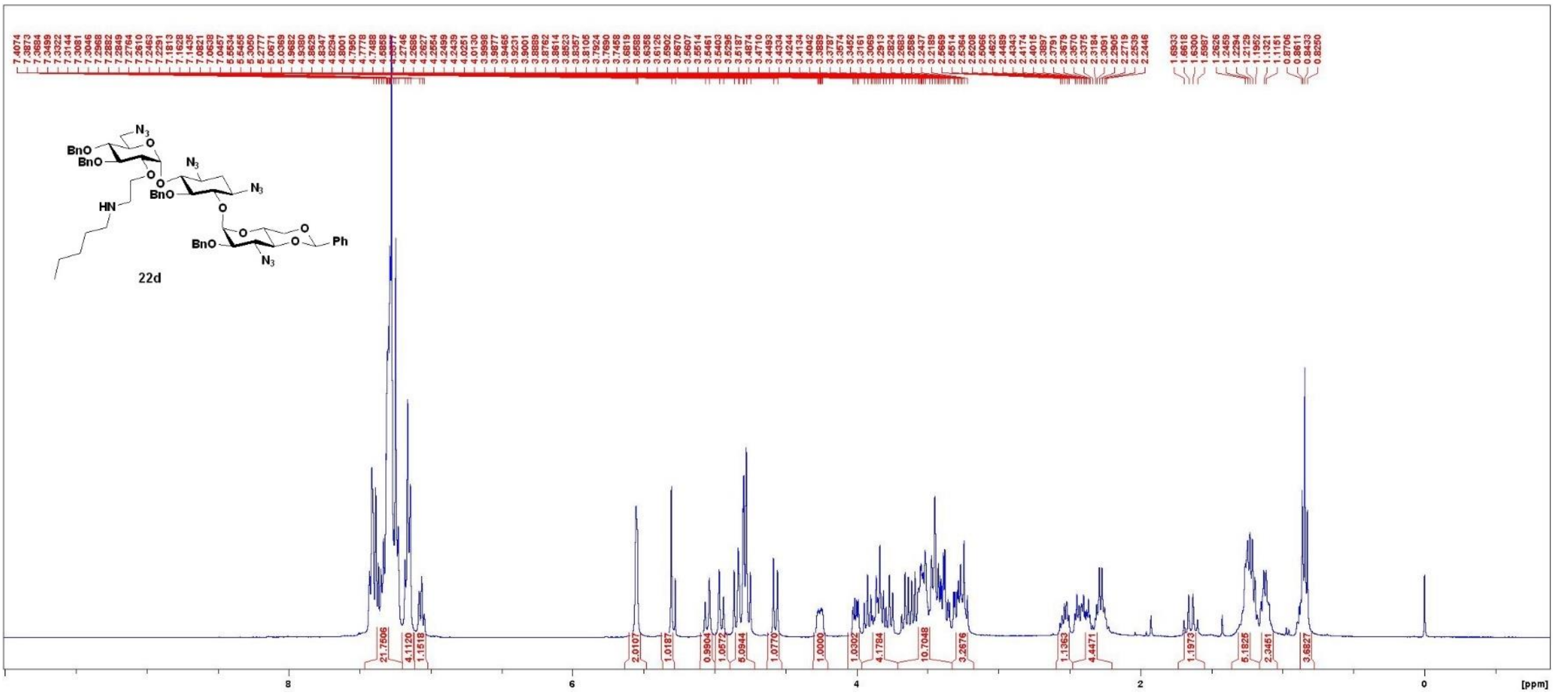

22d
[ppm]

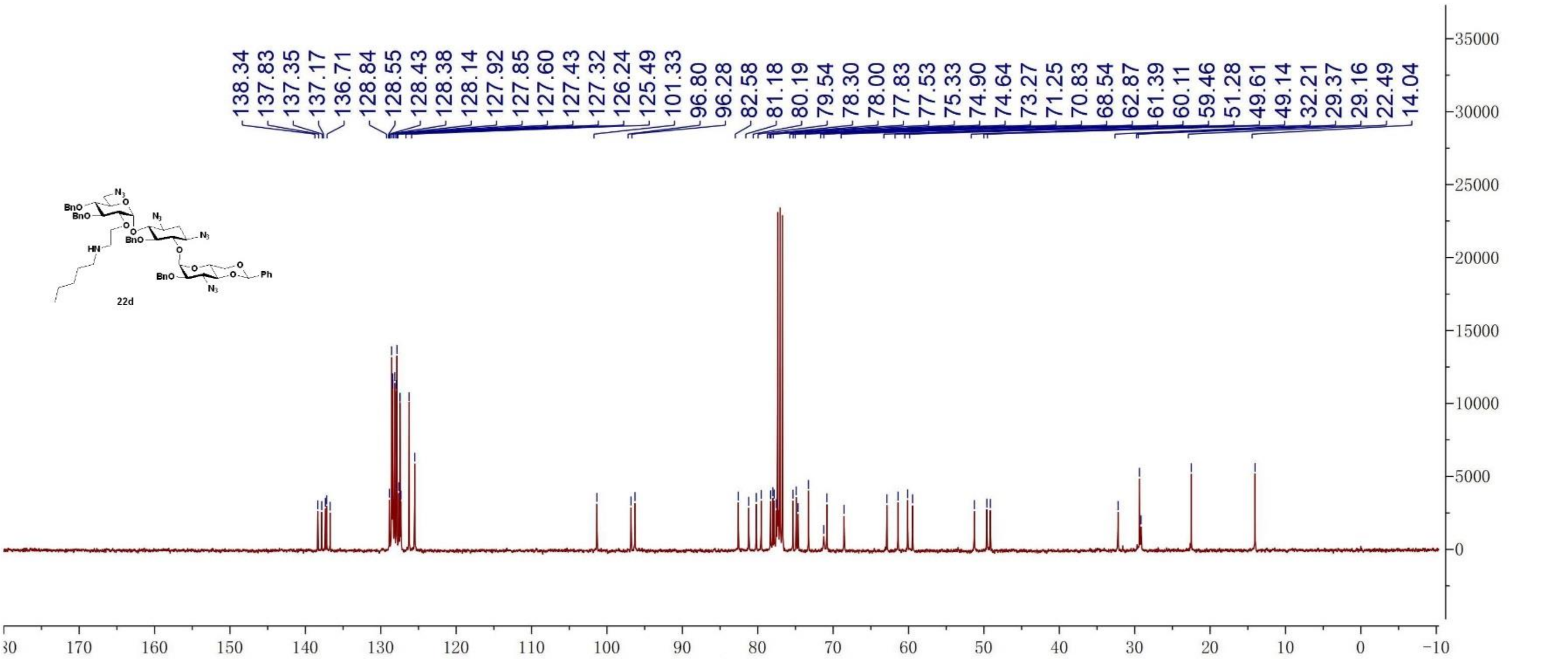

138.34
137.83
137.35
137.17
136.71
128.84
128.55
128.43
128.38
128.14
127.92
127.85
127.60
127.43
127.32
126.24
125.49
101.33
96.80
96.28
82.58
81.18
80.19
79.54
78.30
78.00
77.83
77.53
75.33
74.90
74.64
73.27
71.25
70.83
68.54
62.87
61.39
60.11
59.46
51.28
49.61
49.14
32.21
29.37
29.16
22.49
14.04
22d

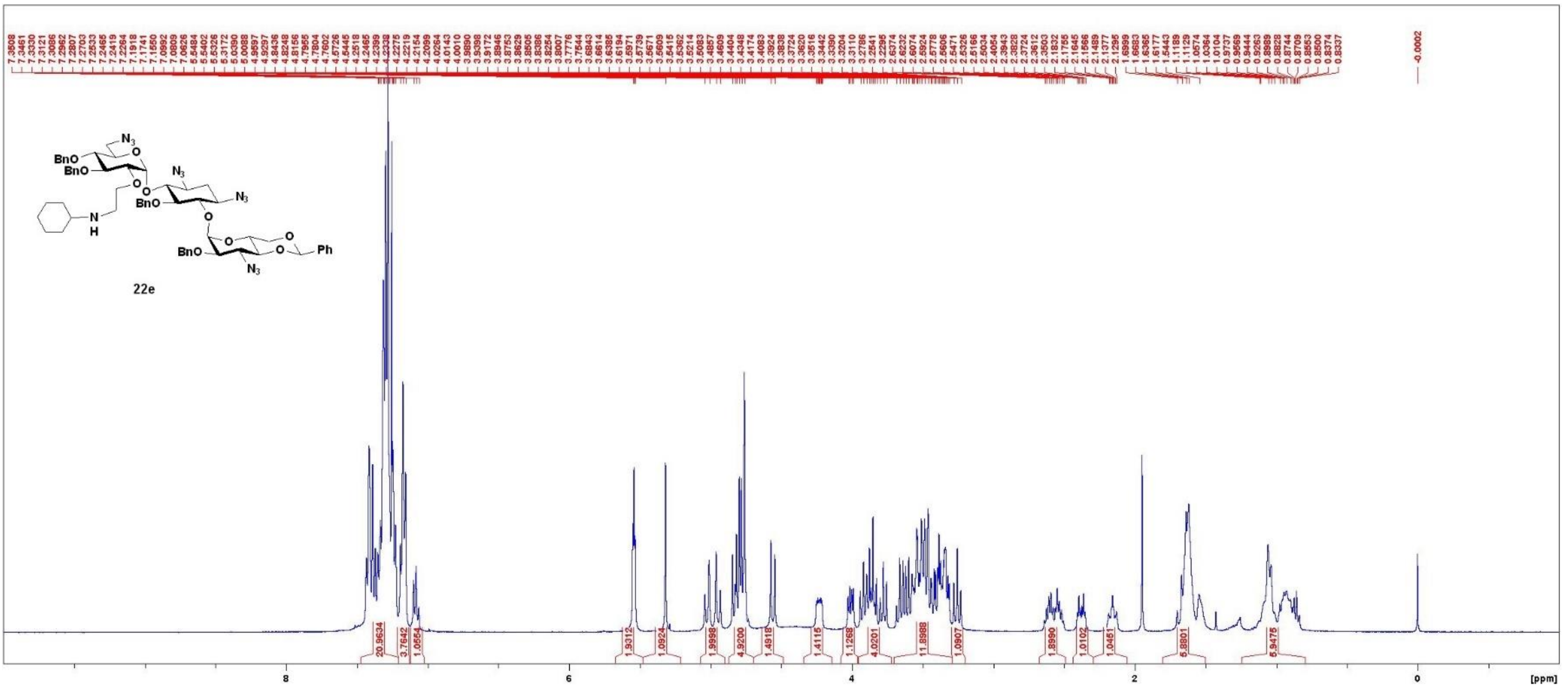

BnO
BnO
N3
N3
BnO
N3
N
H
BnO
N3
Ph
22e
8
6
4
2
0
[ppm]

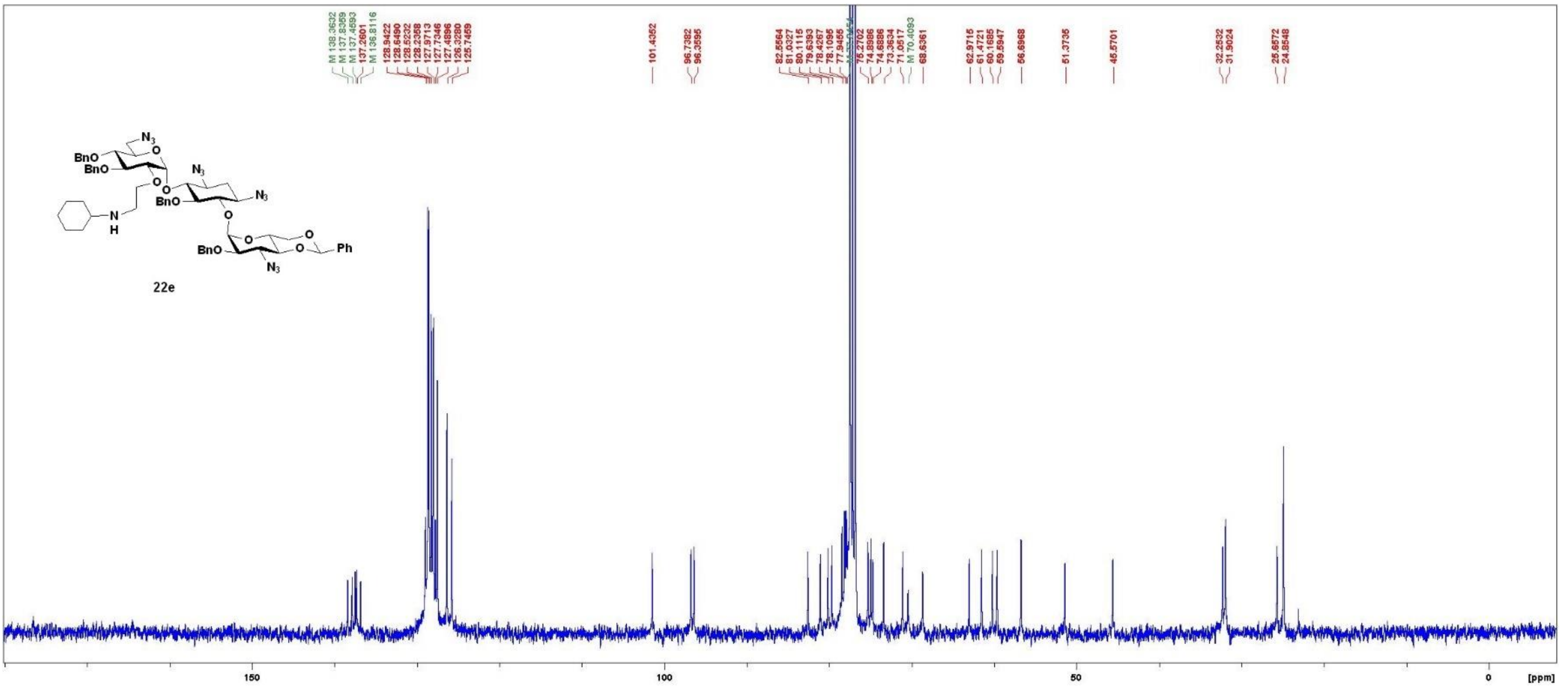

BnO
BnO
N3
N3
BnO
N3
N
H
BnO
N3
Ph
22e
150
100
50
0
[ppm]

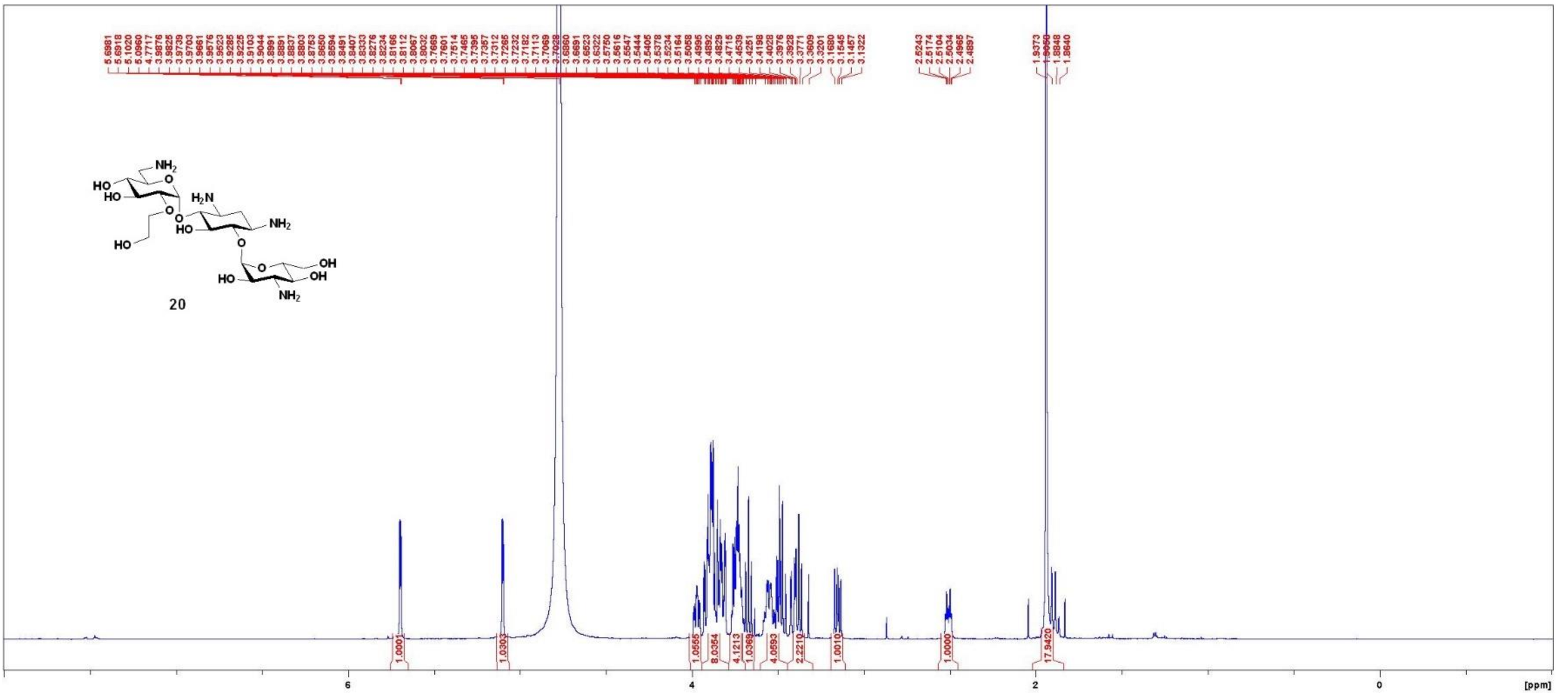
HO
HO
NH2
O
O
H2N
HO
O
NH2
HO
O
O
OH
OH
HO
NH2
20
6
4
2
0
[ppm]

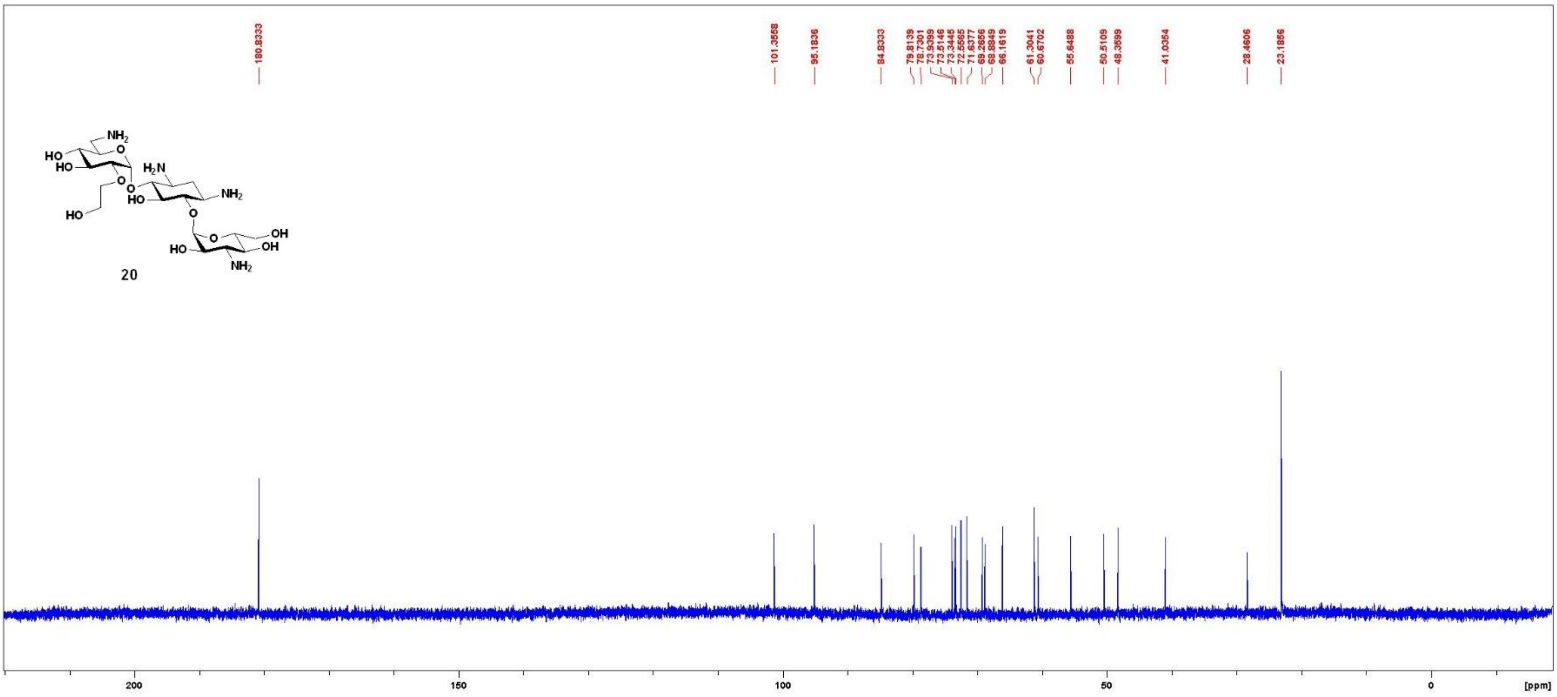
180.8333
101.3558
95.1836
84.8333
79.8139
78.7301
73.9399
73.5146
73.3445
72.5565
71.6377
69.2656
68.8849
66.1619
61.3041
60.6702
55.6488
50.5109
48.3599
41.0354
28.4606
23.1856
20
200
150
100
50
0
[ppm]

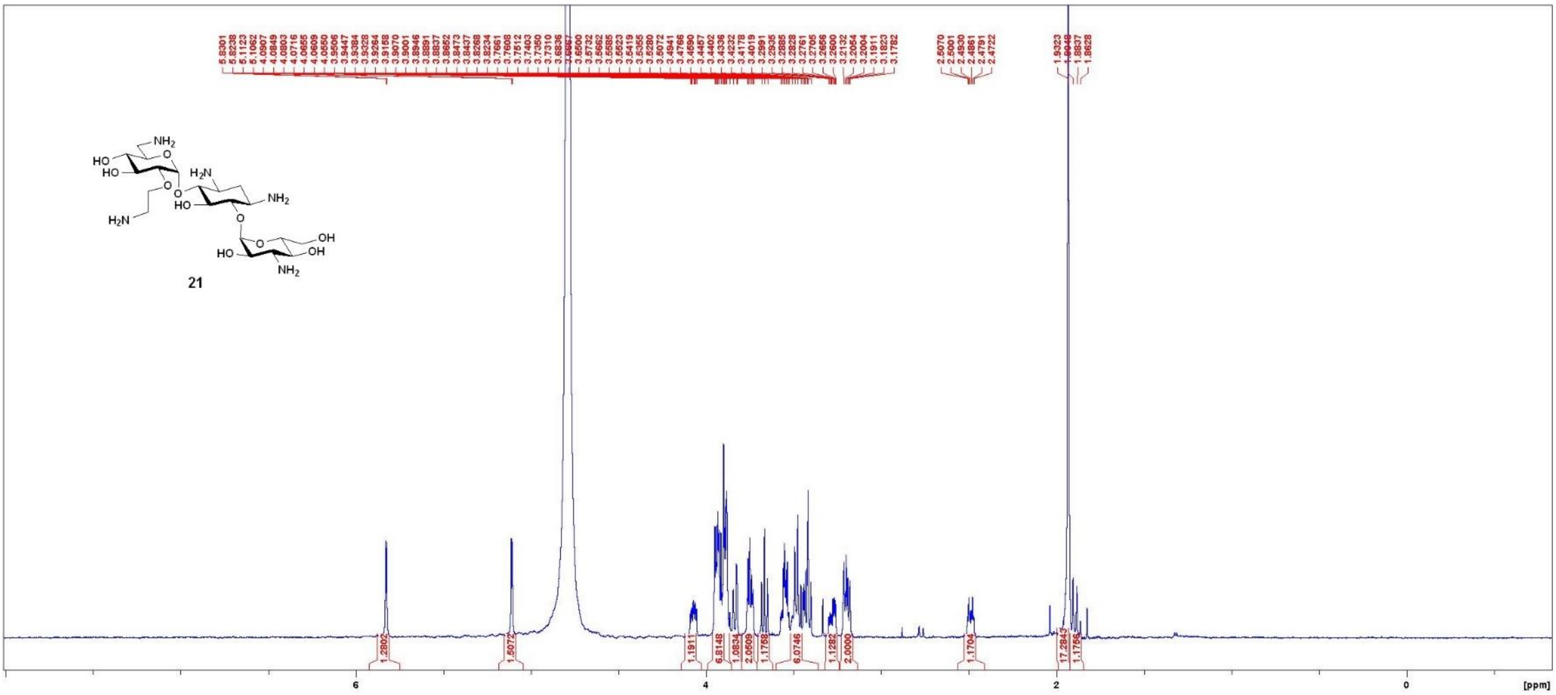

21
[ppm]

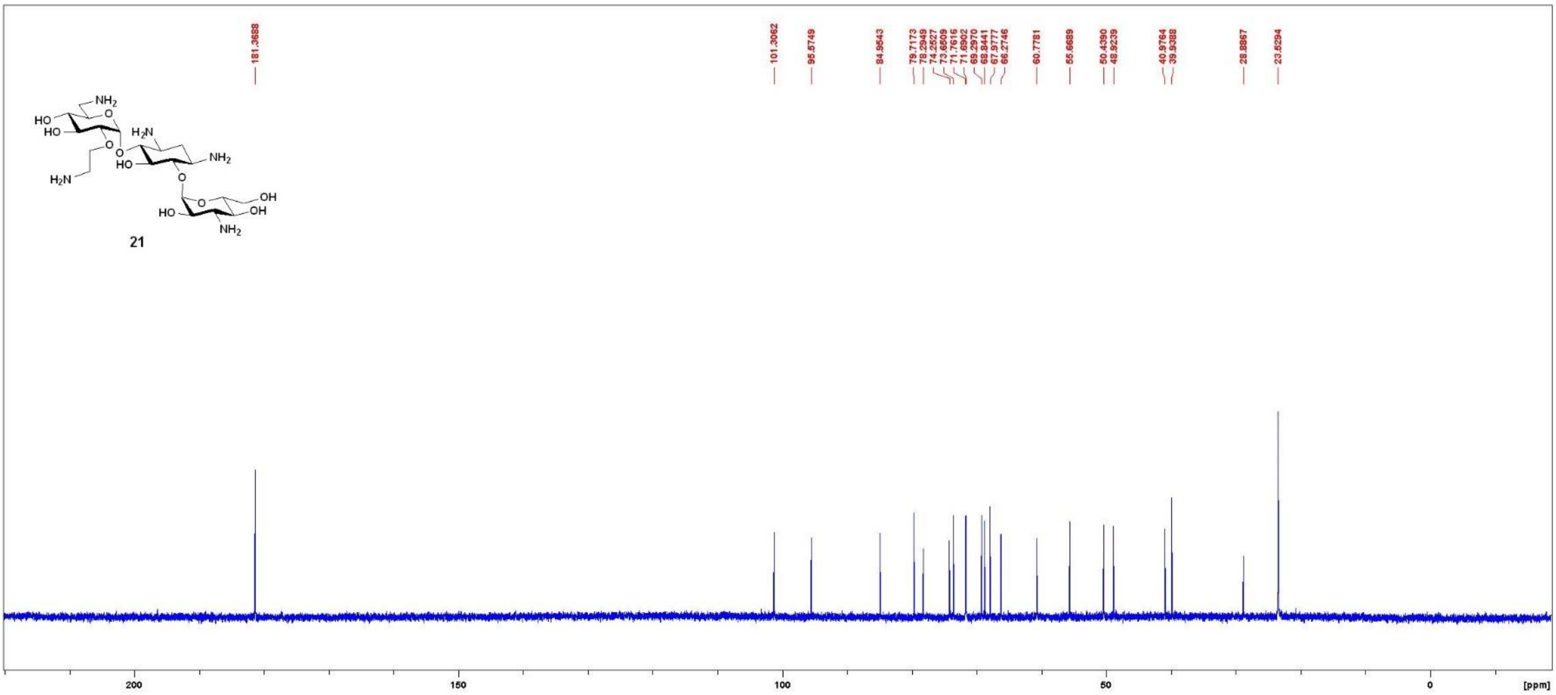

21
181.3688
101.3062
95.5749
84.9543
79.7173
78.2949
74.2527
73.6509
71.7616
71.6902
69.2970
68.8441
67.9777
66.2746
60.7781
55.6689
50.4390
48.9239
40.9764
39.9388
28.8867
23.5294
200
150
100
50
0
[ppm]

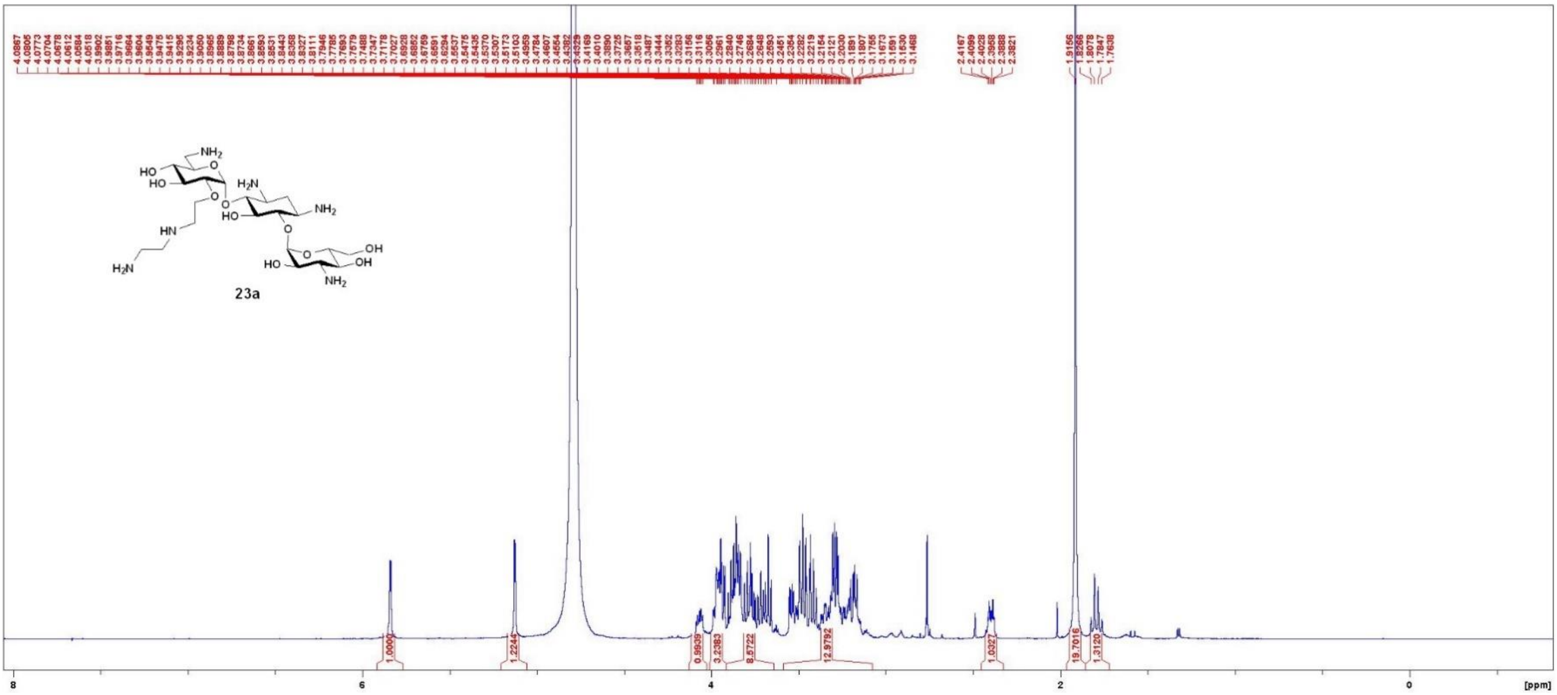
23a
1.0000
1.2244
0.9939
3.2383
8.5722
12.9792
1.0327
19.7016
1.3120
8
6
4
2
0
[ppm]

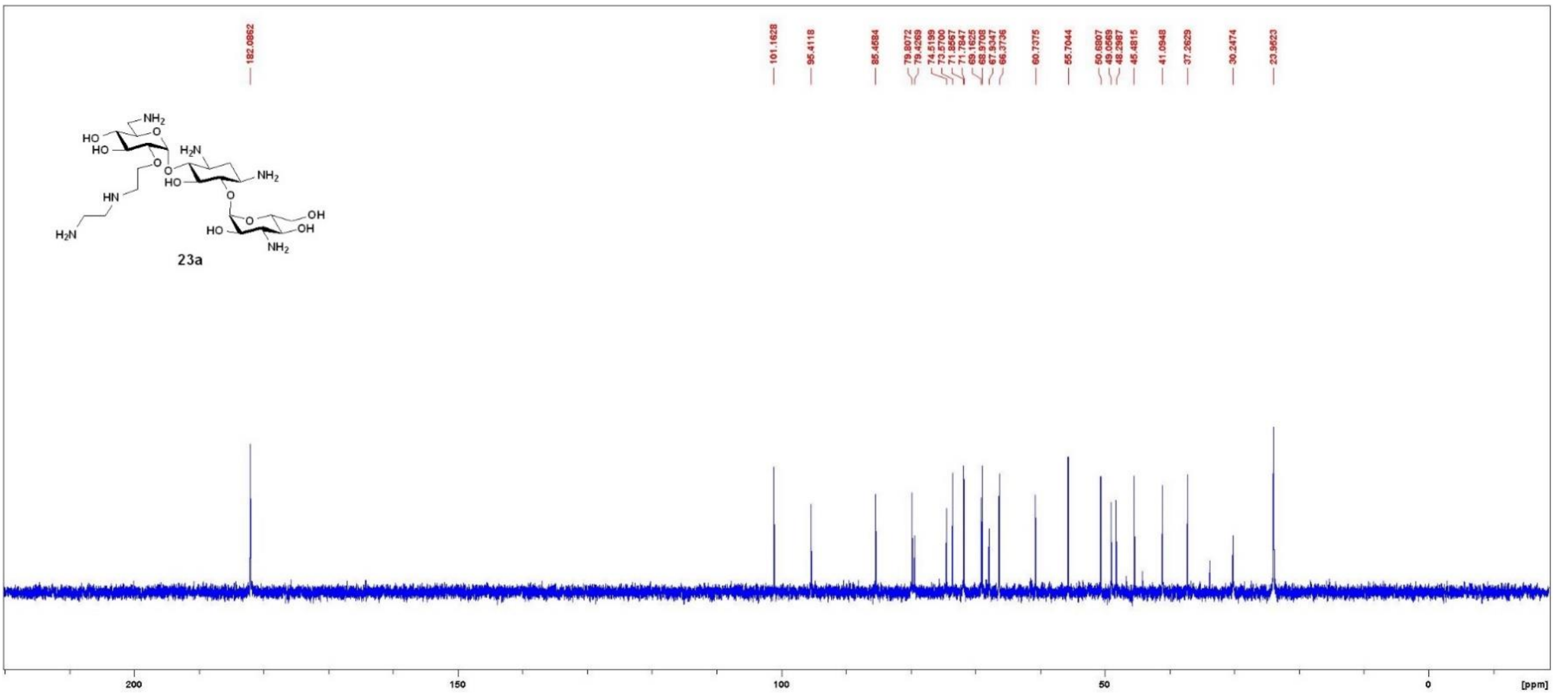
182.0862
101.1628
95.4118
85.4684
79.8072
79.4269
74.5199
73.5700
71.8567
71.7947
69.1625
68.9708
67.9347
66.3736
60.7375
55.7044
50.6907
49.0569
48.2987
45.4815
41.0948
37.2629
30.2474
23.9623
23a
200
150
100
50
0
[ppm]

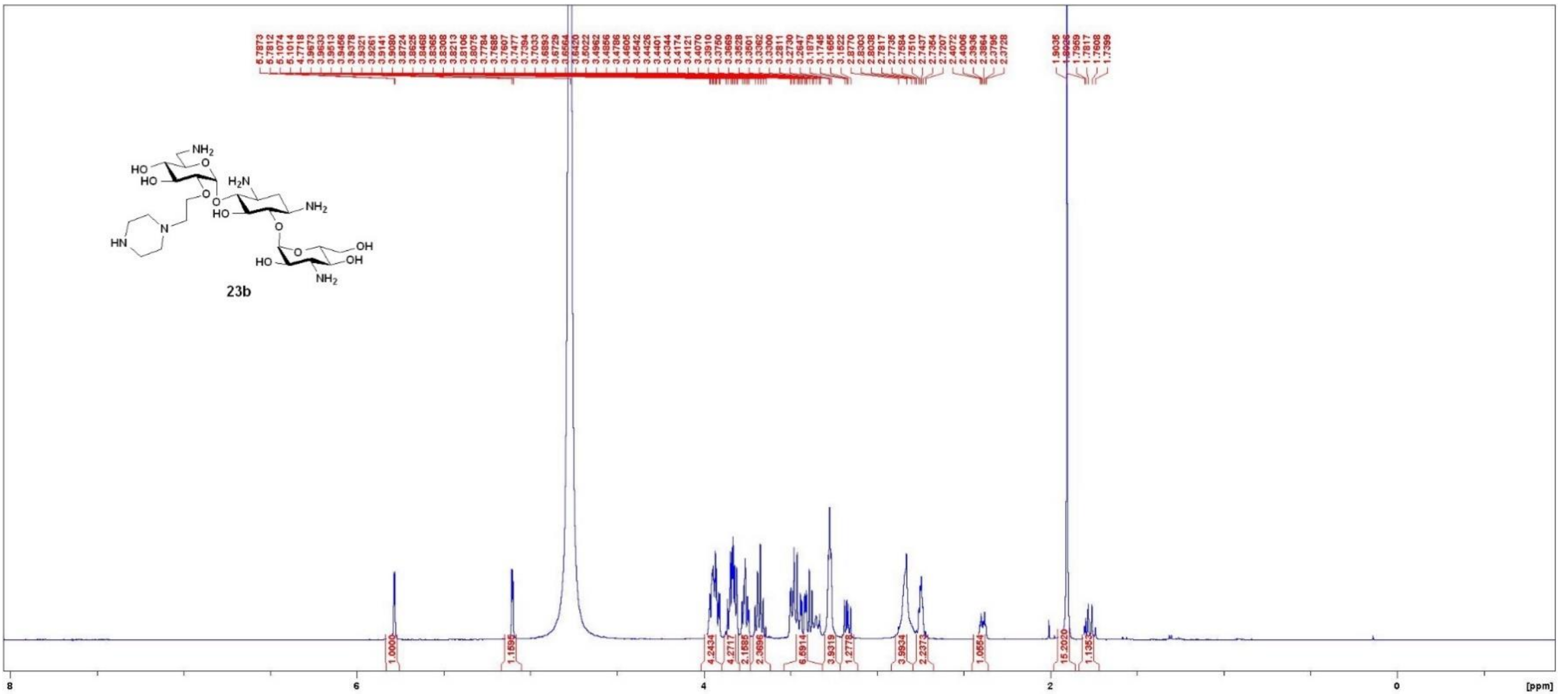

23b
[ppm]

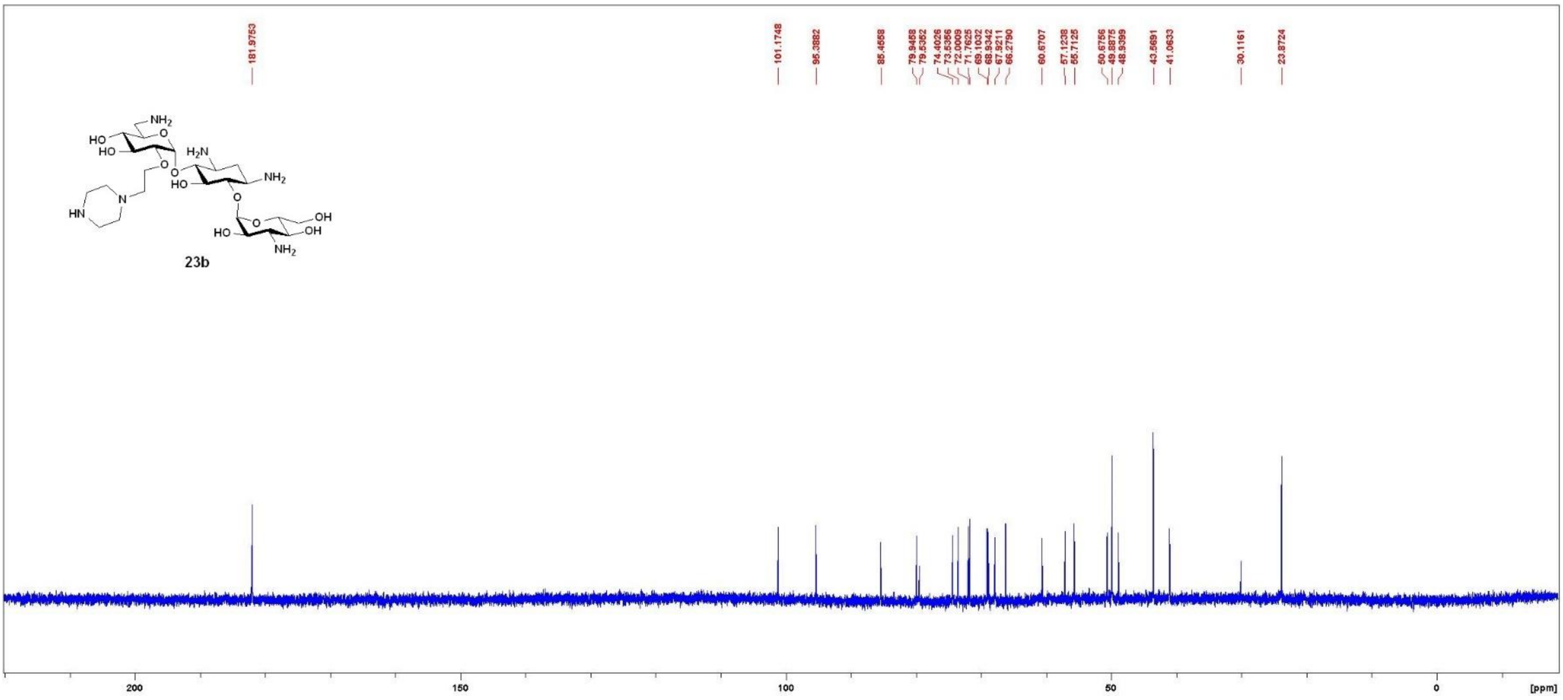

181.9753
101.1748
95.3882
85.4658
79.9458
79.5352
74.4026
73.5256
72.0009
71.7625
69.1032
68.9342
67.9211
66.2790
60.6707
57.1238
55.7125
50.6756
49.8875
48.8399
43.5691
41.0633
30.1161
23.8724
23b
[ppm]

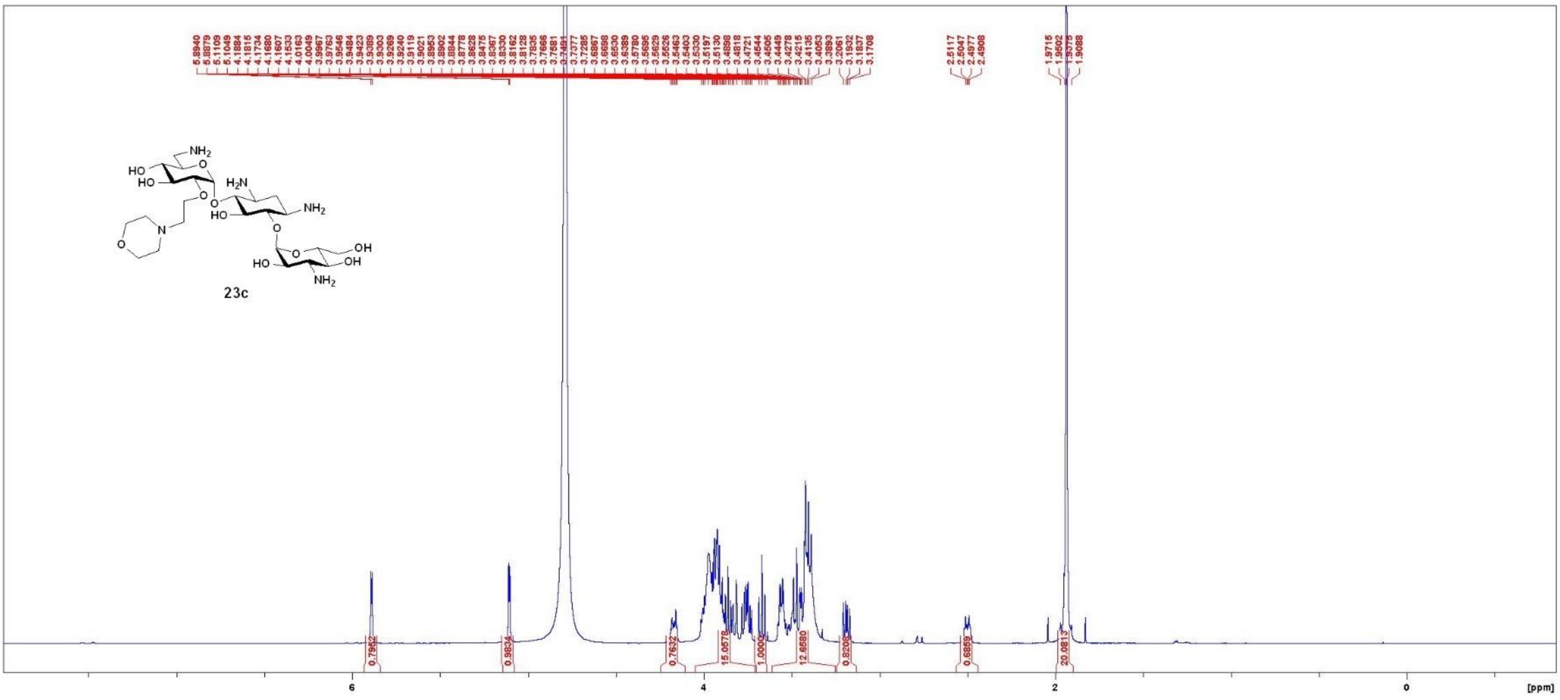
5.8940
5.8879
5.1109
5.1049
4.1884
4.1815
4.1734
4.1680
4.1607
4.1533
4.0163
4.0049
3.9967
3.9763
3.9546
3.9484
3.9423
3.9389
3.9303
3.9269
3.9240
3.9119
3.9021
3.8953
3.8902
3.8844
3.8778
3.8628
3.8475
3.8367
3.8330
3.8162
3.8128
3.7835
3.7666
3.7581
3.7491
3.7377
3.7285
3.6867
3.6698
3.6530
3.6389
3.5780
3.5695
3.5629
3.5526
3.5463
3.5403
3.5330
3.5197
3.5130
3.4898
3.4818
3.4721
3.4544
3.4505
3.4449
3.4278
3.4215
3.4135
3.4053
3.3893
3.2061
3.1932
3.1837
3.1708
2.5117
2.5047
2.4977
2.4908
1.9715
1.9502
1.9379
1.9088
HO
NH2
HO
H2N
O
O
NH2
HO
O
N
O
HO
O
OH
OH
NH2
23c
0.7952
0.9834
0.7632
15.0578
1.0000
12.6580
0.8206
0.6859
20.0813
6
4
2
0
[ppm]

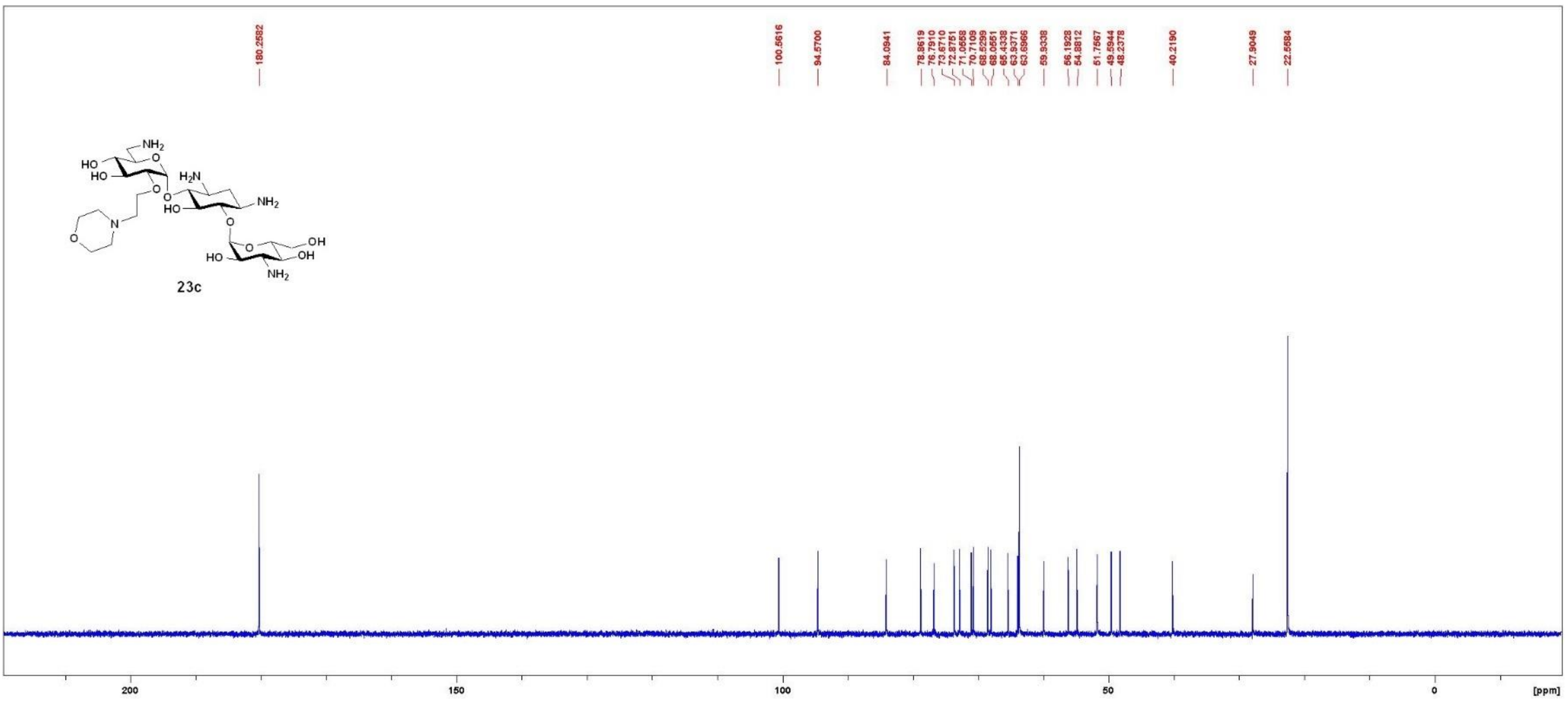
180.2582
100.5616
94.5700
84.0941
78.8619
76.7910
73.6710
72.8751
71.0658
70.7109
68.5299
68.0551
65.4338
63.9371
63.6966
59.9338
56.1928
54.8812
51.7567
49.5944
48.2378
40.2190
27.9049
22.5584
HO
NH2
HO
H2N
O
O
NH2
HO
O
N
O
HO
O
OH
OH
NH2
23c
200
150
100
50
0
[ppm]

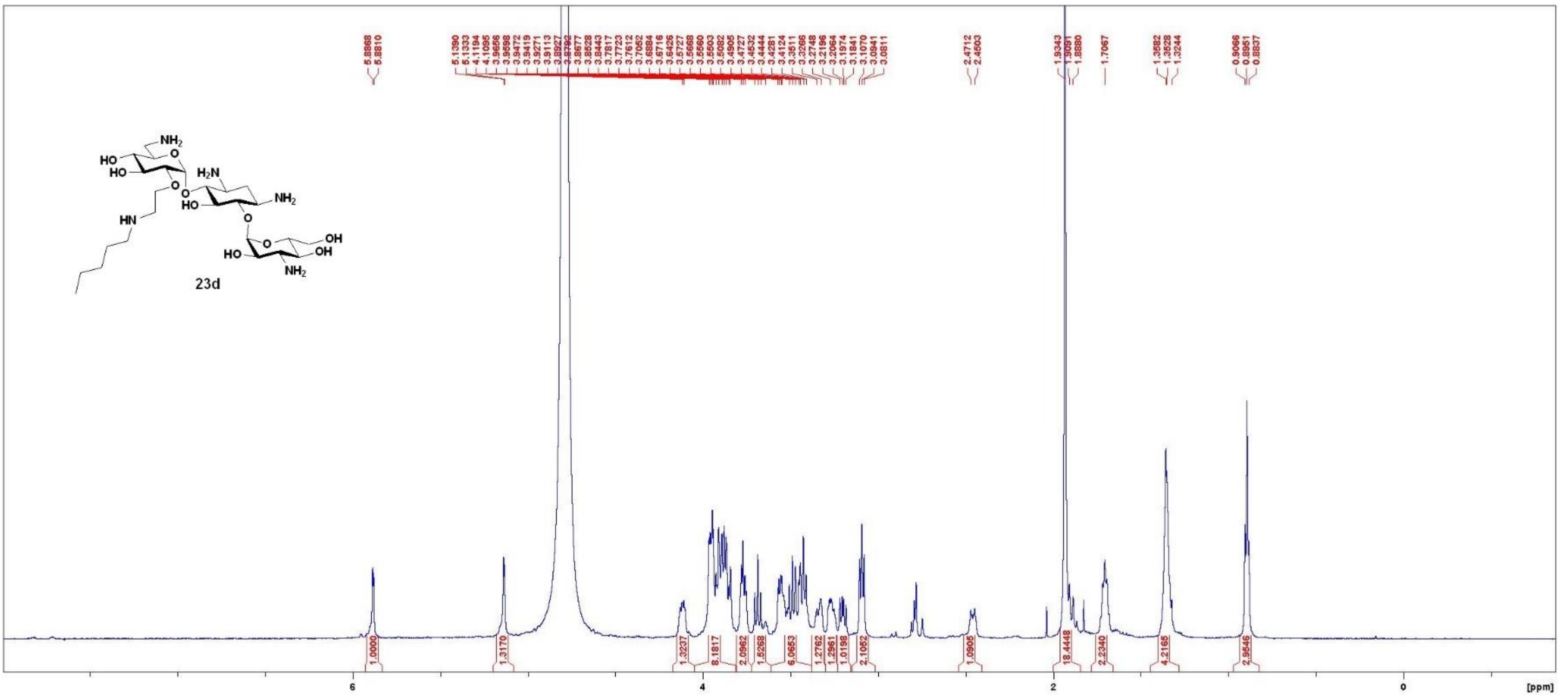
23d
[ppm]

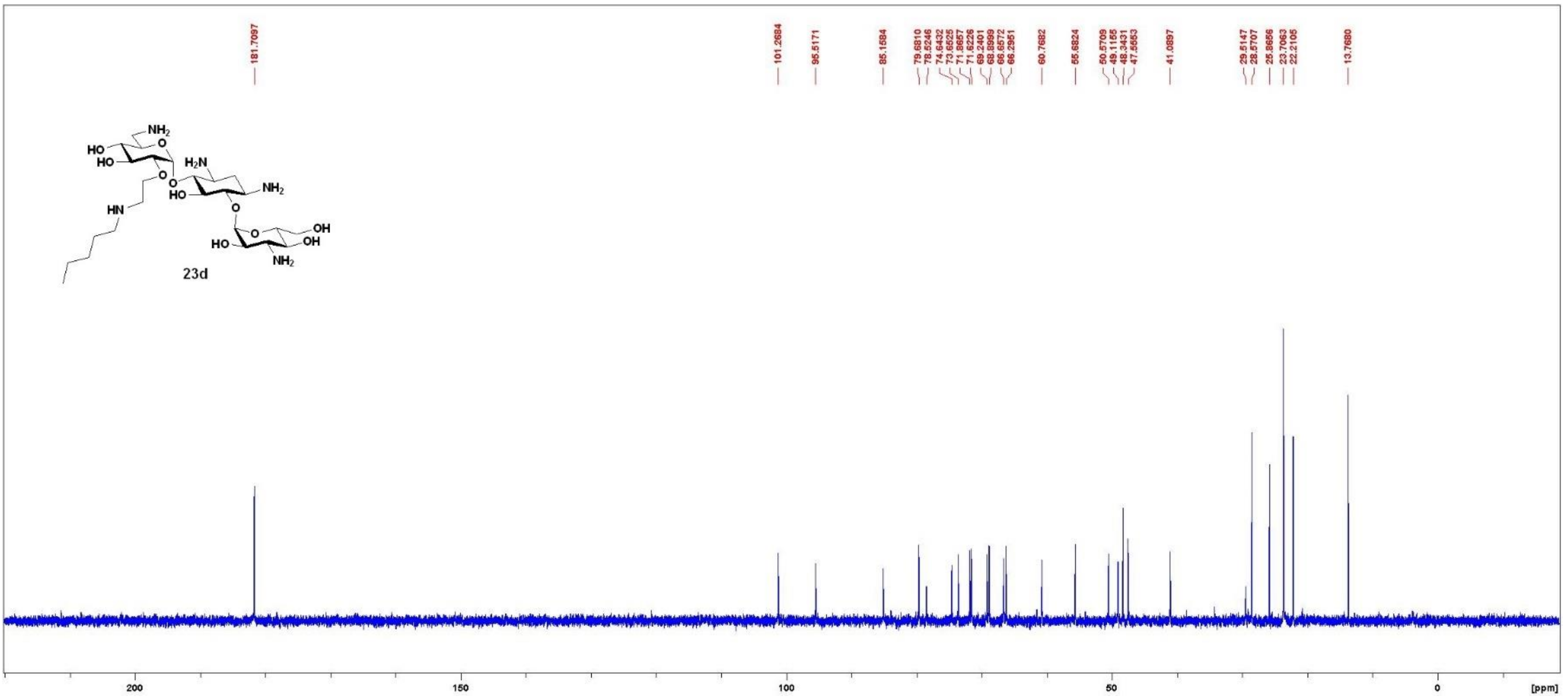
23d
[ppm]

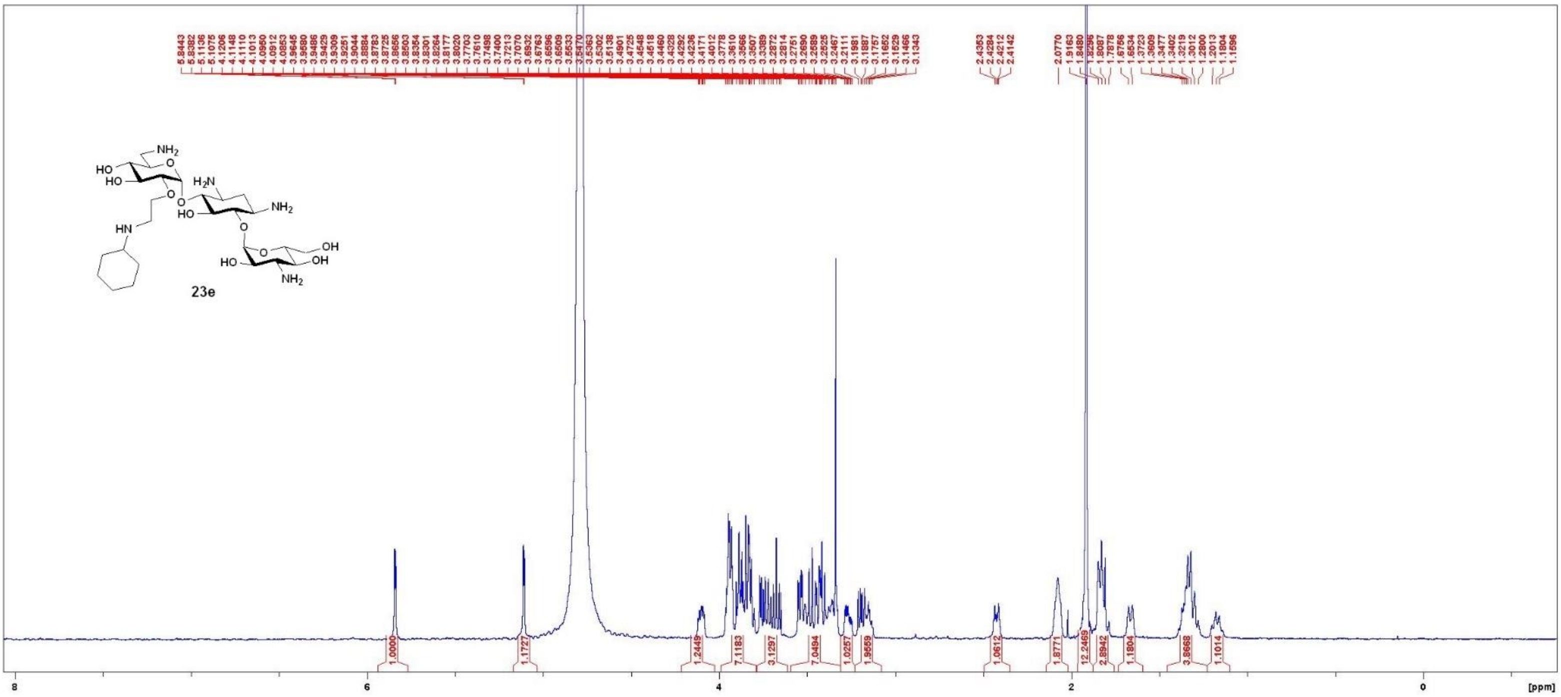
HO
HO
NH2
O
O
HN
H2N
HO
NH2
O
HO
OH
OH
NH2
23e
[ppm]

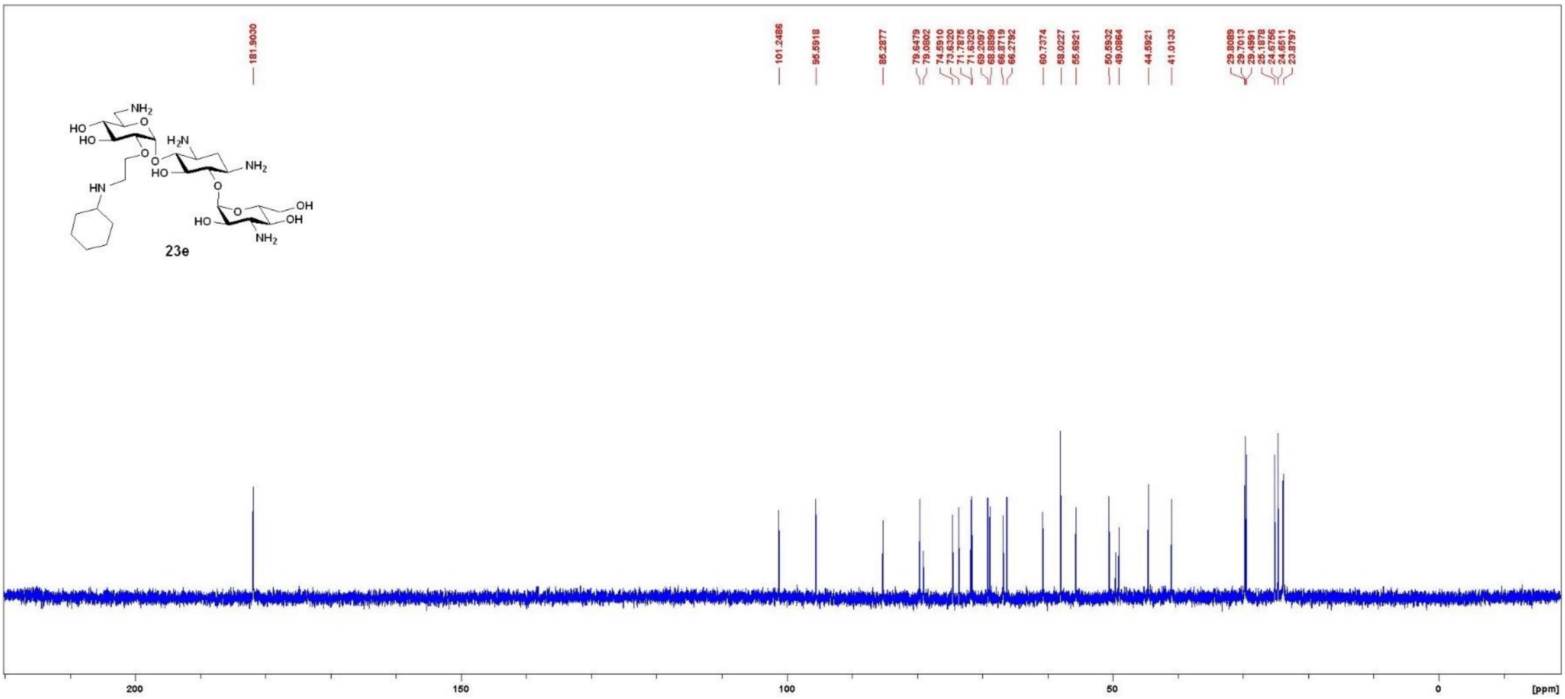
181.9030
101.2486
95.5918
85.2877
79.6479
79.0802
74.5910
73.6320
71.6320
69.2097
68.8899
66.8719
66.2792
60.7374
58.0227
55.6921
50.5332
49.0864
44.5921
41.0133
29.8089
29.7013
29.4991
25.1878
24.6756
24.6511
23.8797
23e
[ppm]
